# Supplementary material for: Collective colony growth is optimized by branching pattern formation in Pseudomonas aeruginosa
Source: Mol Syst Biol. 2021 Apr 26;17(4):e10089. doi: 10.15252/msb.202010089 (PMC8073002; doi:10.15252/msb.202010089)
Supplement: Supplementary file 1 — Appendix [file MSB-17-e10089-s009.pdf]

Table of Contents

Appendix Supplementary Methods: Model development

Appendix Figure S1. Estimating model parameters from growth curves of *Pseudomonas*.

Appendix Figure S2. The mobility of cells is inversely correlated with the agar density of the medium.

Appendix Figure S3. The predicted optimal patterns are consistent in models assuming branch extension in 1D or 2D.

Appendix Figure S4. Using neural networks to accelerate parameter screening.

Appendix Figure S5. Distributions of parameters that generate anti-nutrient-gradient colony growth.

Appendix Supplementary Methods: Model development

I. An optimization model for branched colony growth

We use optimization modeling to find the branching pattern that maximizes colony growth efficiency under given growth conditions. In this optimization model, the parameters of pattern morphology are variables, and the overall biomass accumulation of the colony (i.e., the total cell number) is the objective function. We consider two different formulations of branching patterns: in a simpler scenario when the colony is initiated from a strip under homogeneous growth conditions, branches do not bifurcate and extend along the same direction (1D branch extension); when the colony is initiated from a point inoculum, branches bifurcate as the colony expands and extend towards different directions (2D branch extension).

1. Formulation with 1D branch extension

For a colony initiated from a strip with length R at one end of a rectangular domain Ω with homogeneous growth conditions, we assume it develops parallel, non-bifurcating branches with equal length and uniform branch density that extend towards the other end of the domain (Figure 1C). This pattern is characterized by two parameters: branch width (W) and branch density (D, the number of branches in one unit of length). The total width of all branches is WDR and it cannot be greater than the domain size, hence $WDR \leq R$, or $WD \leq 1$. In the extreme case, when $WD = 1$, the pattern becomes non-branching. Simulations are carried out with patterns with varying W and D.

We develop a model with minimal assumptions that captures the key elements of colony growth: cell growth (multiplication), cell movement, nutrient consumption, and nutrient diffusion.

If nutrient is unlimited or uniform, the shape of colonies makes no difference to colony growth; therefore, nutrient consumption and diffusion are necessary for the model. The distribution of nutrient, $N(\boldsymbol{x}, t)$, is a function of the position ($\boldsymbol{x}$) and time (t) and described by

$$\frac{\partial N}{\partial t} = D_N \nabla^2 N - \beta_N f_G, \tag{1}$$

where D_N is the nutrient diffusivity, β_N is the consumption rate of nutrient, and f_G is a function describing the growth of cells. As shown by experiments (Appendix Figure S1A), the growth curves of *Pseudomonas* cells in swarming media are approximately linear at high cell density, and the growth rate is nutrient-dependent but saturated at high nutrient concentration. We therefore fit the growth curves of cells using the equation

$$f_G = \frac{N}{N+K_N} \frac{C_m}{C+C_m} C, \tag{2}$$

where $C(t)$ is the cell density, which we assume to be uniform within the colony for simplicity, K_N is the half-saturation nutrient concentration of nutrient-dependent growth, and C_m is the half-saturation cell density of cell density-dependent growth. However, the optimality of branching patterns does not depend on this exact form of the growth function.

We describe cell growth and cell movement as two separate steps. In the first step, cell density increases due to cell growth and is described by an ordinary differential equation (see below). In the second step, we consider the change of the colony shape only but not the distribution of cell density. Here, since the branch width and density are predetermined parameters, only the branch length is changing.

Cell growth is described by

$$\frac{\mathrm{d}C}{\mathrm{d}t} = \frac{\int_{\Omega} \alpha_C f_G \mathrm{d}\boldsymbol{x}}{WDRL}, \tag{3}$$

where α_C is the cell growth rate. Here, the total increment of cell density (the amount of cell growth integrated over the area of the entire colony, Ω) is averaged to the total colony area, which is the product of the branch width (W), branch density (D), the domain width (R), and the length of branches ($L(t)$).

If we assume that the energy converted from nutrient is allocated to either cell growth or cell movement in a constant ratio, the amount of energy spent on cell movement, $E(t)$, is proportional to cell growth:

$$\frac{\mathrm{d}E}{\mathrm{d}t} = \int_{\Omega} \alpha_C f_G \mathrm{d}\boldsymbol{x}. \tag{4}$$

We assume that the net expansion of the colony is proportional to the amount of energy allocated to movement, through the production of biosurfactant, the generation of flagella, or other types of mechanisms. Therefore, the increase of colony area is a function of the energy spent per unit time:

$$\frac{\mathrm{d}L}{\mathrm{d}t} WDR = \gamma \frac{\mathrm{d}E}{\mathrm{d}t}. \tag{5}$$

Here, γ is a coefficient relating the amount of energy for cell movement to the expansion rate of the colony and is hitherto referred to as the efficiency of colony expansion. For the same amount of energy, with greater γ, the colony expands faster.

From Equation (4) and (5), we obtain the elongation rate of branches

$$\frac{\mathrm{d}L}{\mathrm{d}t} = \frac{\gamma}{WDR} \int_{\Omega} \alpha_C f_G \mathrm{d}\boldsymbol{x}. \tag{6}$$

The initial nutrient concentration is uniform and given by N_0. The initial branch length is l_0. Simulations with different patterns are initiated with the same total number of cells, i.e., $C = c_0/A$, where c_0 is a constant representing the initial biomass and A is the initial area of the colony. We enforce no-flux boundary conditions for the nutrient.

2. Formulation with 2D branch extension

When a colony is initiated from a single point inoculum at the center of a plate, it develops branches that bifurcate as the colony expands (Figure 4A). We still describe the pattern with branch width (W) and branch density (D). In this scenario, the growth directions of the branches follow the local nutrient gradient. The local branch density of a branch is given by $1/d$, where d is the distance of the branch tip to its nearest neighbor; the branch bifurcates to maintain the local branch density around D. Specifically, we track the tip of a growing branch and calculate the local branch density. As the branch extends, if the local branch density falls below a predefined threshold, branch bifurcation is triggered and a new subbranch initiates. Because the local branch density doubles after a bifurcation event, the threshold branch density for bifurcation we choose is $\frac{2}{3}D$, so that the local branch density oscillates between $\frac{2}{3}D$ and $\frac{4}{3}D$.

We describe nutrient distribution, cell growth, and branch extension using similar equations as described above:

$$\frac{\partial N}{\partial t} = D_N \nabla^2 N - \beta_N f_G, \qquad (7)$$

$$\frac{\mathrm{d}C}{\mathrm{d}t} = \frac{\int_{\Omega} \alpha_C f_G \mathrm{d}\boldsymbol{x}}{\int_{\Omega} \mathrm{d}\boldsymbol{x}}, \tag{8}$$

$$\frac{\mathrm{d}L_i}{\mathrm{d}t} = \frac{\gamma}{W_i} \int_{\Omega_i} \frac{1}{n} \alpha_C f_G \mathrm{d}\boldsymbol{x}, \tag{9}$$

$$f_G = \frac{N}{N + K_N} \frac{C_m}{C + C_m} C. \tag{10}$$

Here, $\Omega_i(t)$ is the i-th branch and n is the number of branches sharing the same location $\boldsymbol{x}$.

The initial nutrient concentration is uniform and given by N_0. Patterns are initialized as a small circle at the center of the domain with a radius of r_0. The initial cell density $C = c_0/(2\pi {r_0}^2)$. The initial number of branch tips is $2\pi r_0 D$. We enforce no-flux boundary conditions for the nutrient.

II. A coarse-grained model for predicting colony patterns based on the optimization rule

Using the optimization model, we establish a mapping between the optimal branch properties (W and D) and growth conditions (N_0 and γ). This mapping allows us to predict colony patterns under heterogenous growth conditions (which require the formulation with 2D branch extension): given the distributions of the initial growth conditions, $N_0(\boldsymbol{x})$ and $\gamma(\boldsymbol{x})$, we obtain the local branch properties, $W(\boldsymbol{x})$ and $D(\boldsymbol{x})$, through interpolation using the mapping. Hence, a branch at position $\boldsymbol{x}_0$ has a width of $W(\boldsymbol{x}_0)$ and a density of $D(\boldsymbol{x}_0)$. Nutrient distribution, cell growth, and branch extension are calculated using Equations (7)-(10). The initial and boundary conditions are the same as in the optimization model with 2D branch extension described above.

III. Parameters

We estimate the parameters from experimental measurements as described below. In simulations, the parameters are optimized within the ranges given by the estimations, so that the simulated patterns are the most consistent with experimental observations.

In 1.5% agar gel, at 20°C, the diffusivity of molecules is roughly inversely correlated with the molecular weight: glucose (180 g/mol): 6×10^{-10} m^2/s; sucrose (342 g/mol): 4.2×10^{-10} m^2/s (Schantz & Lauffer, 1962); phthalocyanine dye (895 g/mol): $(2.5\pm0.2)\times10^{-10}$ m^2/s (Støren *et al.*, 2003). The majority of Bacto casamino acids has a molecular weight less than 250 g/mol. The diffusivity of molecules in liquids is proportional to the absolute temperature according to the Stokes–Einstein equation. The diffusion of small molecules changes very little when agar density is < 1% (Ratzke & Gore, 2016). Therefore, the diffusivity of casamino acids in 0.5% agar at 37°C is at least 5.5×10^{-10} m^2/s = 2 mm^2/h.

Fitting the growth equations in liquid

$$\frac{\mathrm{d}N}{\mathrm{d}t} = -\beta_N f_G, \quad (11)$$

$$\frac{\mathrm{d}C}{\mathrm{d}t} = \alpha_C f_G, \quad (12)$$

$$f_G = \frac{N}{N+K_N}\frac{C_m}{C+C_m}C, \quad (13)$$

with the growth curves of *Pseudomonas* in liquid swarming medium (Appendix Figure S1A), we have $\alpha_C = 0.8$ h^{-1}, $K_N = 0.8$ g L^{-1} and $C_m = 0.05$ c.u. (c.u.: cell density unit).

The ratio of α_C and β_N is equal to the cells generated and the nutrient consumed at a certain timepoint. In a liquid swarming medium with 4 g/L casamino acids, the final cell density reaches OD_{600} of 0.4. Therefore, α_C/β_N = (0.4 c.u.)/ (4 g/L) and $\beta_N = 8$ g L^{-1} h^{-1} c.u.$^{-1}$.

γ represents how much the colony is able to expand per unit amount of energy. The absolute value of γ is difficult to measure, but since all other parameters are determined, we can estimate γ by fitting with experimental results. On a swarming medium with 0.5% agar and 8 g/L casamino acids, it takes 24 h for colonies to develop stripe patterns with W = 3.8 mm, D = 0.10 mm^{-1}, and L = 25.6 mm. Fitting these data gives us γ = 5 mm h^{-1} c.u.$^{-1}$ on 0.5% agar.

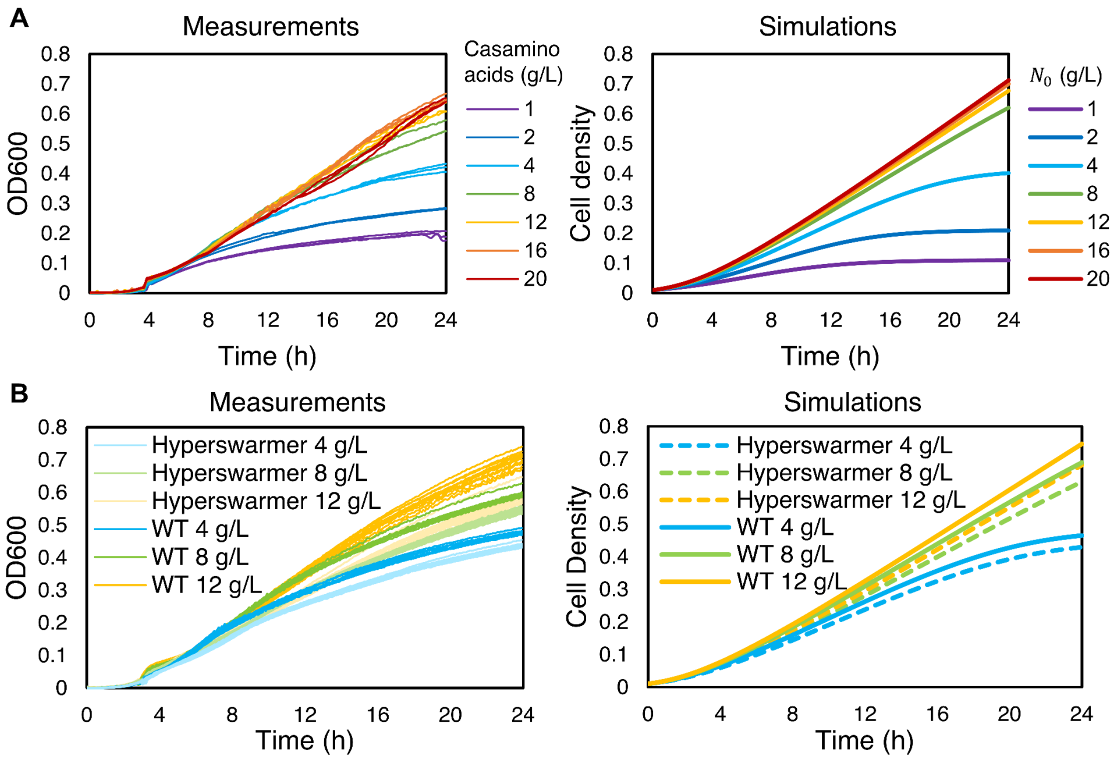

Appendix Figure S1. Estimating model parameters from growth curves.

A. The growth function (f_G in the model) and the parameters are determined by fitting with the growth curves of *Pseudomonas*. Left: Measurements of cell densities of *Pseudomonas* in liquid swarming media containing different concentrations of casamino acids (3 replicates for each concentration). In each well of a 96-well plate, we add 200 μl liquid swarming media, 2 μl cell culture (overnight cultures of each sample were diluted 10x~50x to the same cell density), and 50 μl mineral oil. The cells were then incubated in a plate reader at 37 °C and OD_{600} measurements were taken at 10-min intervals for 24 hours. Background signals measured from media containing no cells were subtracted from the data. Right: Simulations using equations describing nutrient (N) and cell density (C) in liquid phase: $\frac{\mathrm{d}N}{\mathrm{d}t} = -\beta_N f_G, \frac{\mathrm{d}C}{\mathrm{d}t} = \alpha_C f_G$, where the growth function is $f_G = \frac{N}{N+K_N}\frac{C_m}{C+C_m}C$,

with parameters $\alpha_C = 0.8$ h^{-1}, $\beta_N = 8$ g L^{-1} h^{-1} c.u.$^{-1}$, $K_N = 0.8$ g/L, and $C_m = 0.05$ c.u.. Different curves represent results with different initial nutrient concentrations (N_0).

B. The growth rate of the hyperswarmers is slightly lower than that of the wild-type (WT). Left: Measurements of cell densities of wild-type or hyperswarmers in liquid swarming media containing different concentrations of casamino acids (12 replicates for WT and 14 replicates for hyperswarmers with each nutrient concentration). See panel A for details of measurements. Right: Fitting the growth curves by simulations using the same equations as in panel A, with parameters $\alpha_C = 0.94$ h^{-1} for wild-type and $\alpha_C = 0.87$ h^{-1} for hyperswarmers, $\beta_N = 8$ g L^{-1} h^{-1} c.u.$^{-1}$, $K_N = 0.8$ g/L, and $C_m = 0.045$ c.u.. Therefore, the growth rate of the hyperswarmers is 7% lower than the wild-type. Moreover, the decrease in the biomass accumulation of the hyperswarmers under nutrient-deficient condition (casamino acids 4 g/L) in the liquid medium is less than 10%, which is significantly lower than that on a solid medium.

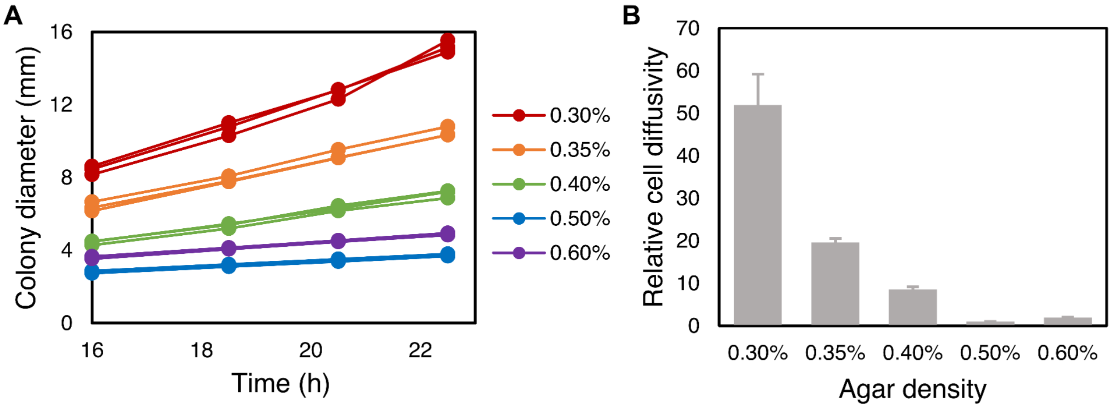

Appendix Figure S2. The mobility of cells is inversely correlated with the agar density of the medium.

Assuming the growth and expansion of a radially expanding colony can be described by Fisher's equation, the colony advances as a traveling wave with a wave speed proportional to $\sqrt{D}$, where D is the diffusivity of cells (Murray, 2007). Therefore, we can estimate the cell diffusivity by measuring the size of colonies.

A. The diameters of colonies linearly increase with time with different speeds on different agar densities. *Pseudomonas* colonies were grown on solid LB media, so they expand radially without developing branches. The diameters of the colonies were measured under a microscope (3 replicates for each agar density).

B. Relative cell diffusivities are inversely correlated with the agar densities. We obtain the growth speed, v, of each colony by fitting the data in panel A to linear functions of time. Since the cell diffusivities are proportional to v^2, we can obtain the relative cell diffusivities on different agar densities. Error bars show standard error of the mean.

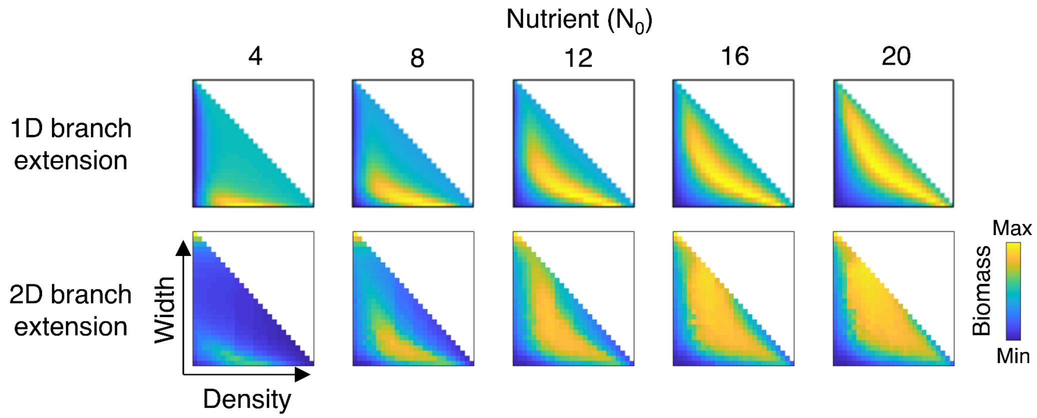

Appendix Figure S3. The predicted optimal patterns are consistent in models assuming branch extension in 1D or 2D.

The optimal patterns predicted by the branch-pattern model implemented with 2D branch extension are similar to those predicted by the strip-pattern model implemented with 1D branch extension. Each subplot shows, with different initial nutrient concentration (N_0), the total biomass at the same time point when varying the branch width and density. At or beyond the diagonal of the heatmap, the colony becomes uniform with no branches. Colors indicate the total biomass scaled to the min/max values.

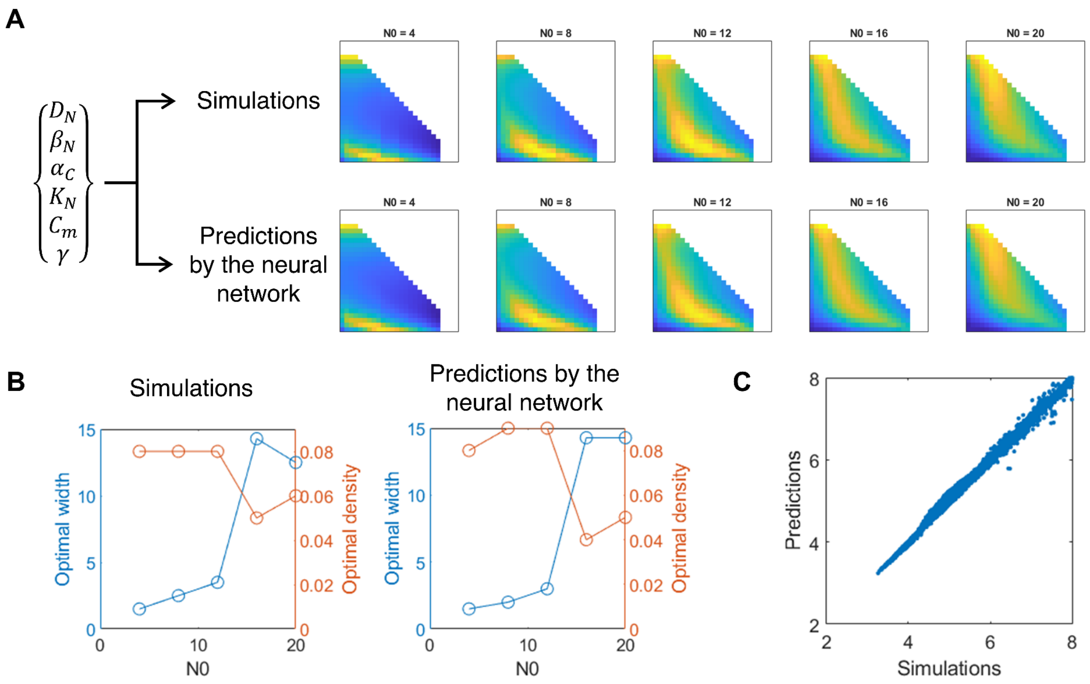

Appendix Figure S4. Using neural networks to accelerate parameter screening.

A, B. Predicting the optimal colony patterns with the optimization model or the neural networks. Results obtained by the model and the neural networks are highly consistent. The results of one set of parameters are shown as an example.

A. For each set of randomized parameters $(D_N, \beta_N, \alpha_C, K_N, C_m, \gamma)$, we calculate the biomass accumulation using the model with the 2D branch extension formulation or the neural networks with 5 different initial nutrient concentrations (N_0) and 351 different combinations of branch width (W) and branch density (D). Colors indicate the total biomass scaled to the min/max values in each subpanel.

B. Using the biomass data obtained with the optimization model or the neural networks, we find the optimal branch width and density at each N_0. By implementing the mapping between the nutrient concentrations and the optimal patterns in the model, we predict the colony patterns growing on a nutrient gradient.

C. Accuracy of the neural networks. Biomass predicted by the neural networks plotted against the biomass obtained by the optimization model for the test data sets. The test sample size is 17,550, generated with 10 sets of randomized parameters, 5 different initial N_0, and 351 different combinations of branch width and density. We used root mean squared errors to evaluate the differences between data generated by the optimization model and data generated by the neural networks: $R^2 = 0.99861$.

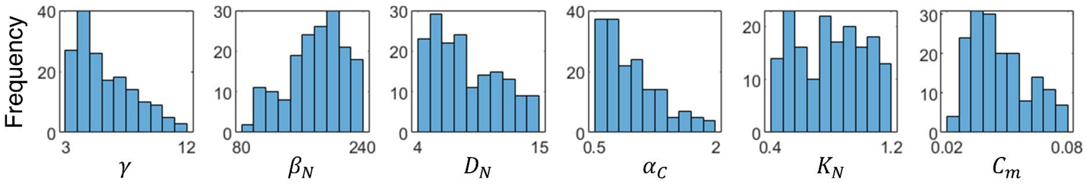

Appendix Figure S5. Distributions of parameters that generate anti-nutrient-gradient colony growth.

We screened through 30,000 parameter sets and identified 918 that generated patterns resembling the anti-nutrient-gradient growth observed in experiments. Histograms show the frequency distribution of each parameter in the identified parameter sets. The distribution of the identified parameters reveals that for anti-nutrient-gradient growth to occur, the nutrient consumption rate (β_N) needs to be relatively high, while the cell growth rate (α_C) and expansion efficiency (γ) need to be relatively low.

References

Murray JD (2007) *Mathematical biology: I. An introduction*. Springer Science & Business Media,

Ratzke C, Gore J (2016) Self-organized patchiness facilitates survival in a cooperatively growing Bacillus subtilis population. *Nat Microbiol* 1: 16022

Schantz EJ, Lauffer MA (1962) Diffusion measurements in agar gel. *Biochemistry* 1: 658-63

Støren T, Simonsen A, Løkberg OJ, Lindmo T, Svaasand LO, Røyset A (2003) Measurement of dye diffusion in agar gel by use of low-coherence interferometry. *Optics letters* 28: 1215-7
