# Supplementary material for: Collective colony growth is optimized by branching pattern formation in Pseudomonas aeruginosa
Source: Mol Syst Biol. 2021 Apr 26;17(4):e10089. doi: 10.15252/msb.202010089 (PMC8073002; doi:10.15252/msb.202010089)
Supplement: Supplementary file 2 — Expanded View Figures PDF [file MSB-17-e10089-s004.pdf]

Expanded View Figures

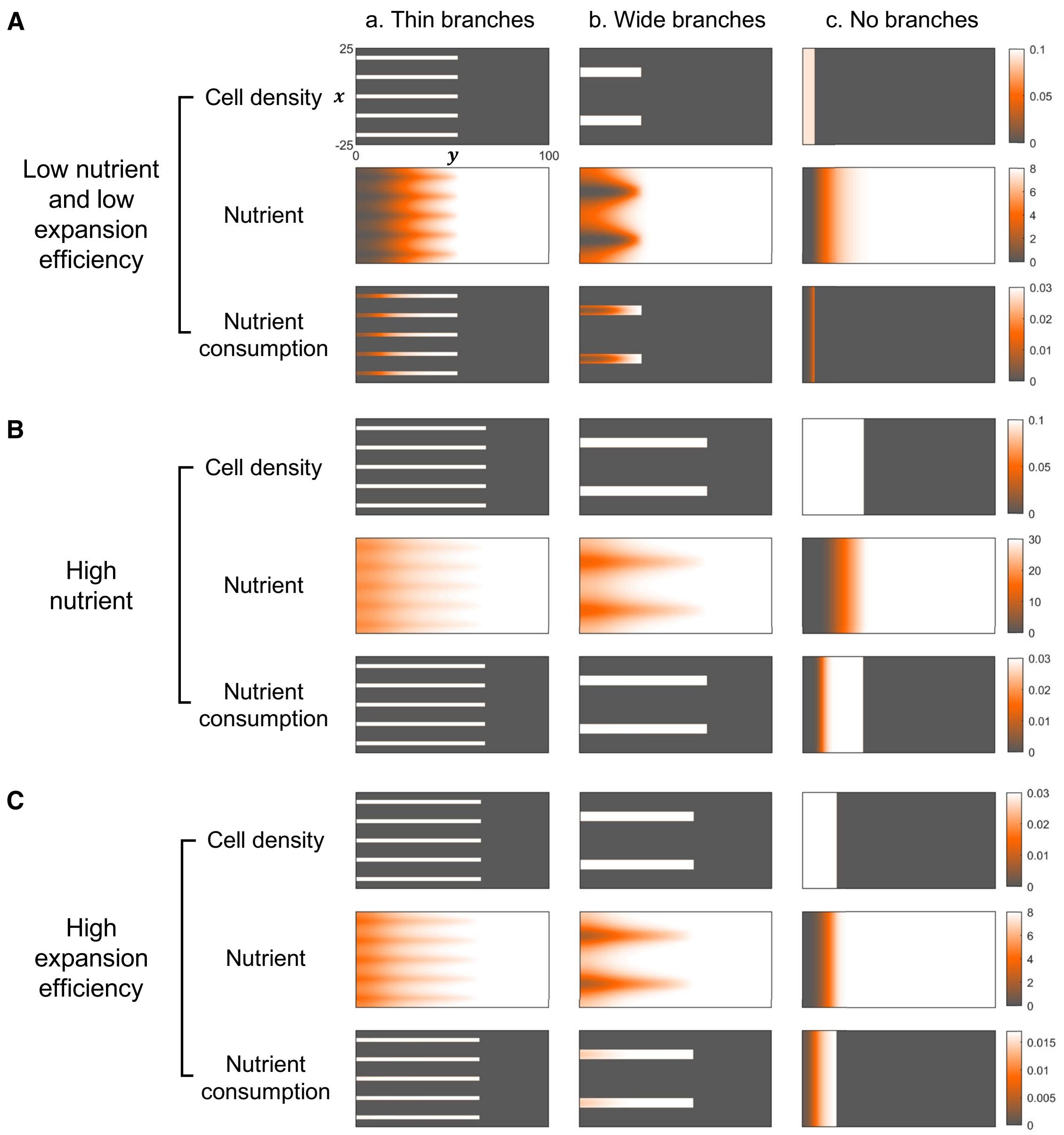

Figure EV1. Simulations reveal how different conditions lead to different optimal colony patterns.

Simulations reveal how the spatial–temporal dynamics of colonies vary depending on the patterns of the colonies and the growth conditions. Left: thin branches (branch width: 2 mm, branch density: 0.10/mm); middle: wide branches (branch width: 5 mm, branch density: 0.04/mm); right: no branches. For different colony patterns, the distributions of cell density (c.u.), nutrient concentration (g/l), and the consumption rate of nutrient (g/l/h) at the same time points (A. t = 20 h, B. t = 18 h, C. t = 9 h) are shown.

A When nutrient is scarce and the expansion efficiency is low (N_0 = 8 g/l, γ = 7.5 mm/h/c.u.), nutrient is quickly depleted in the colony-covered regions and the utilization of nutrient is mainly at the colony front and edges. In this case, the total amount of nutrient utilization is correlated with the length of the colony boundaries, which is higher in colonies with thin branches but minimized in non-branching colonies.

B, C When nutrient is abundant (N_0 = 30 g/l, γ = 7.5 mm/h/c.u.) or the expansion efficiency is high (N_0 = 8 g/l, γ = 25 mm/h/c.u.), colonies expand before consuming all the nutrient in the area covered by cells. Therefore, the consumption of nutrient is also related to the colony area, which is higher in colonies expanding uniformly.

© 2021 The Authors

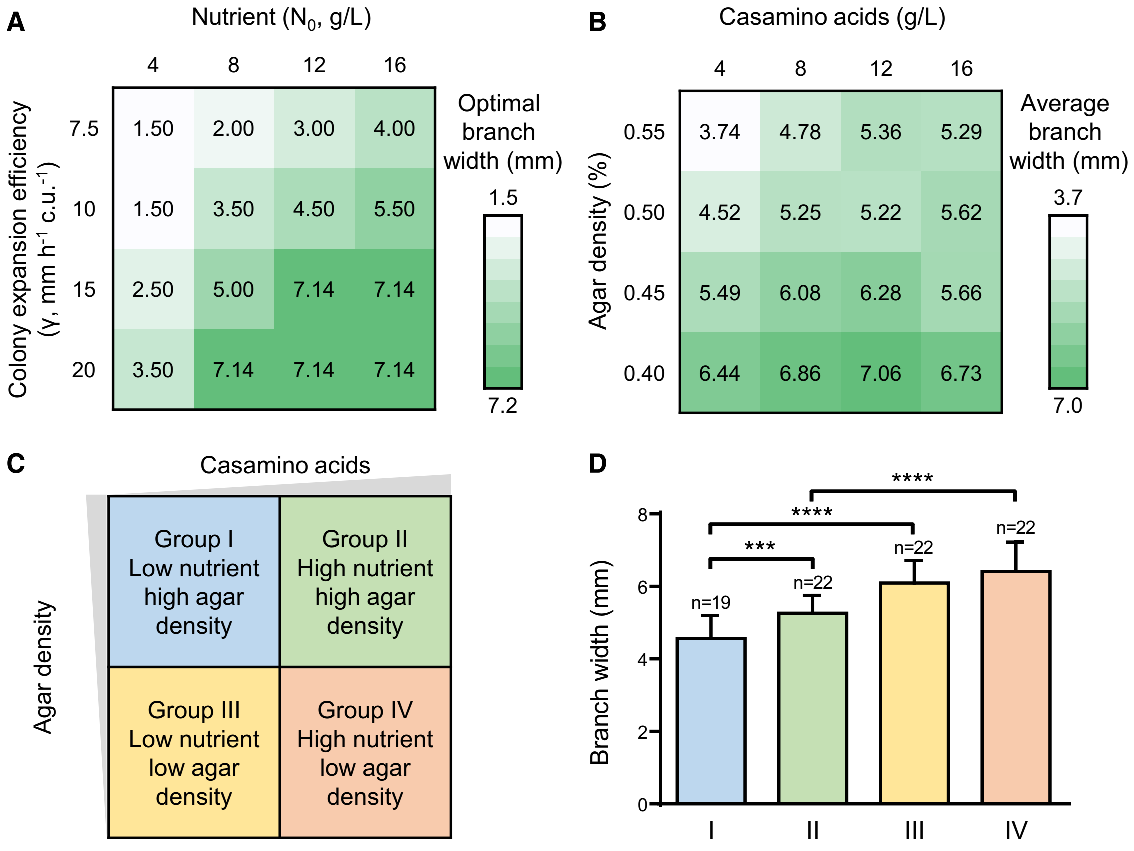

Figure EV2. Branch width of *Pseudomonas* colonies increases with increasing nutrient concentration and decreasing agar density.

A The optimal branch widths (mm; shown by numbers and colors in the table) predicted by the optimization model with different combinations of environmental parameters.

B The average branch widths (mm; shown by numbers and colors in the table) of *Pseudomonas* colonies under different combinations of growth conditions. The mean branch width of a colony was measured in a semi-automated manner (described in detail in Methods and Protocols). For each condition, the average of the mean branch widths of 2–4 colonies was shown.

C The growth conditions of *Pseudomonas* colonies are divided into four groups: I. Low nutrient concentration (4–8 g/l casamino acids) and high agar density (0.50–0.55%); II. High nutrient concentration (10–16 g/l casamino acids) and high agar density (0.50–0.55%); III. Low nutrient concentration (4–8 g/l casamino acids) and low agar density (0.40–0.45%); and IV. High nutrient concentration (10–16 g/l casamino acids) and low agar density (0.40–0.45%).

D The average branch width of each group in (C). The sample size is the number of colonies being measured in each group. Error bars show standard deviations. Unpaired, two-sided *t*-tests were used to compare between groups: I versus II: ***P = 0.0004; I versus III: ****P < 0.0001; II versus IV: ****P < 0.0001 (with Welch's correction because their variances are not significantly different); III versus IV: P = 0.1485.

Source data are available online for this figure.

© 2021 The Authors

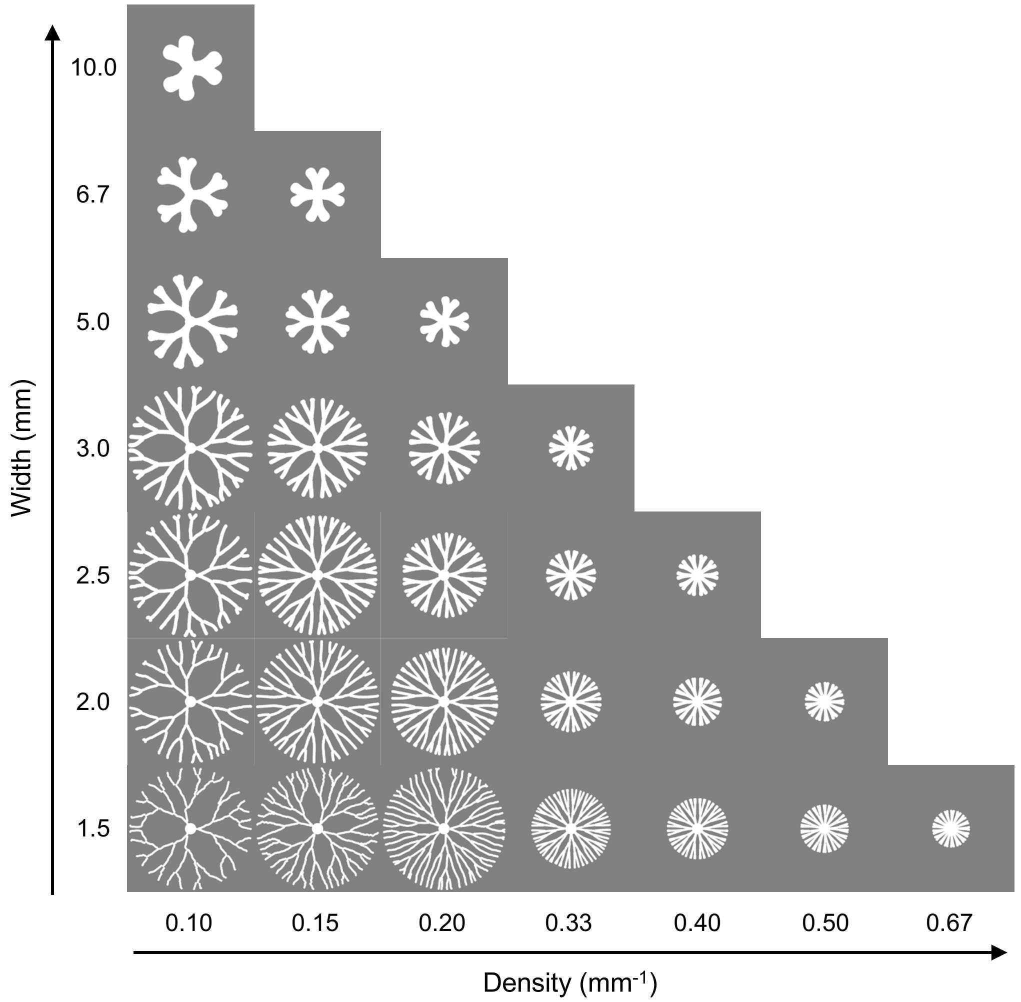

Figure EV3. Simulated patterns with different combinations of branch widths and densities.

Patterns with 2D branch extension generated by the model when different combinations of branch widths and densities are implemented. Other parameters of the model are kept the same (D_N = 9 mm^2/h; β_N = 160 g/l/h/c.u.; α_C = 1.2/h; K_N = 0.8 g/l; C_m = 0.05 c.u.; γ = 7.5 mm/h/c.u.; N_0 = 8 g/l). The patterns are initialized from a disk at the center with uniform initial cell density C_0 = 0.5 c.u. The boundary of a branch is set by the given branch width, and bifurcations are determined by the given branch density. Each branch extends following the local nutrient gradient.

Source data are available online for this figure.

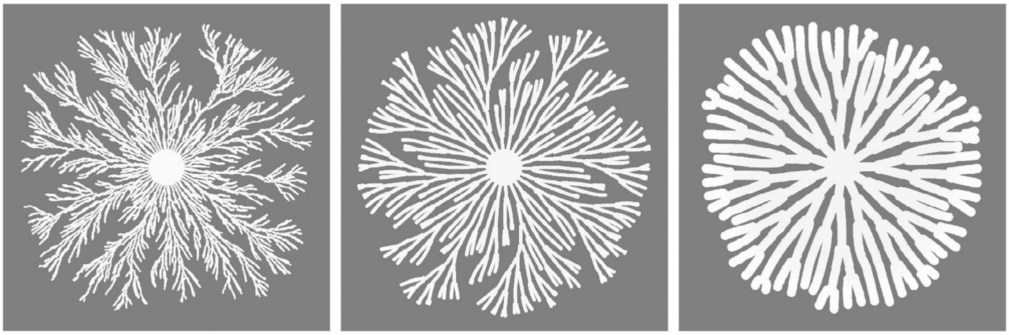

Figure EV4. Simulating diverse colony patterns using the model based on the optimization rule.

Varying parameters of the model results in diverse colony patterns with distinct quantitative features, such as thin dendrites and patterns with densely packed fingers. The parameters used for producing these examples are as follows: D_N = 9.326 mm^2/h; β_N = 152.9 g/l/h/c.u.; α_C = 1.878/h; K_N = 0.8466 g/l; C_m = 0.06050 c.u.; γ = 7.385 mm/h/c.u.; N_0 = 4 g/l (left), 5 g/l (middle), or 8 g/l (right). The branch density and width implemented in the model are the optimal ones that result in maximum biomass accumulation efficiency, as found by optimization modeling using the 2D branch extension formulation.

Source data are available online for this figure.

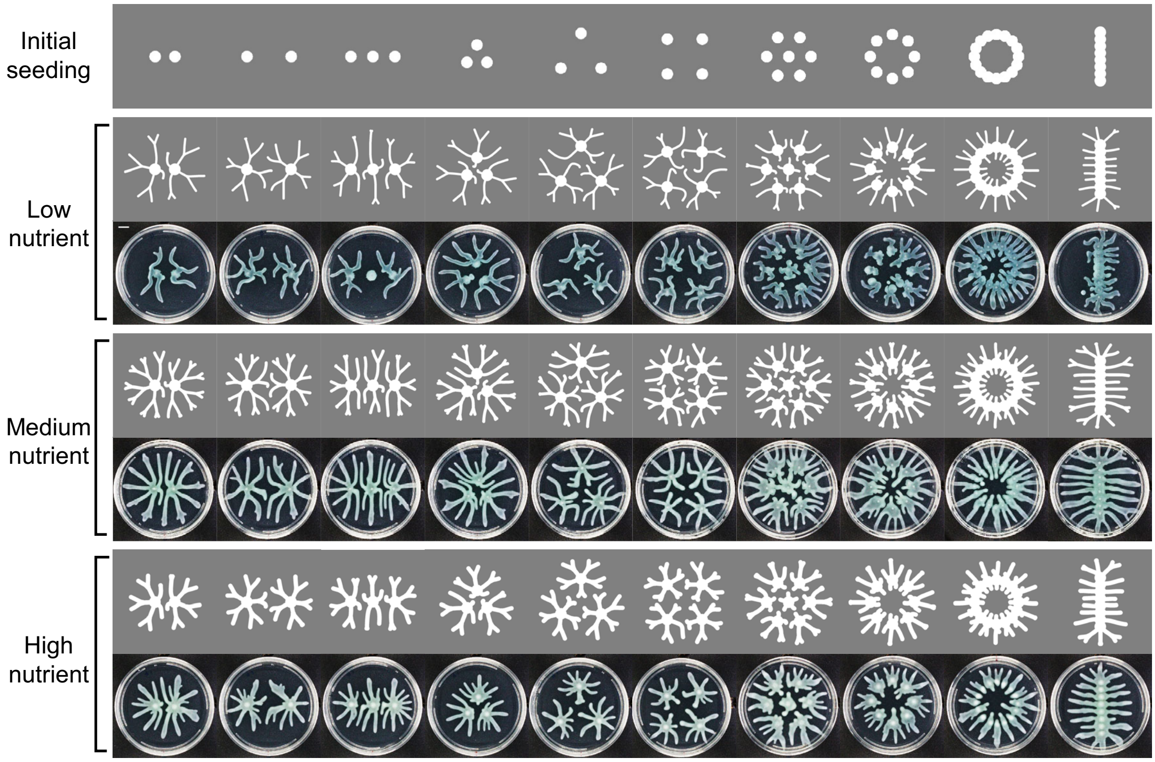

Figure EV5. Predicting colony patterns with various seeding configurations and nutrient concentrations.

The model is able to predict colony patterns with complex initial configurations and different nutrient concentrations. Top row: the initial seeding locations; lower rows: simulated and observed colony patterns with corresponding initial seeding configurations growing on media with different nutrient concentrations (casamino acid concentration: 4, 8, and 16 g/l from top to bottom). In experiments, multiple colonies were seeded on the same petri dish using a MANTIS automated liquid handler (0.1 µl cell culture with OD_{600} ~0.2 was dispensed at each spot). Images are representatives of 2 replicates. Images from Fig 6 and one panel from Fig 1B are reused in the "Medium nutrient" panels. Scale bar: 1 cm. In simulations, patterns are initialized from spots with a radius = 5 mm and uniform initial cell density C_0 = 1.6. The parameters for the simulations are as follows: D_N = 5.749 mm^2/h; β_N = 195.5 g/l/h/c.u.; α_C = 1.105/h; K_N = 0.6635 g/l; C_m = 0.07890 c.u.; γ = 4 mm/h/c.u.; N_0 = 8.5 g/l, 14.5 g/l, or 16.5 g/l from top to bottom. The branch density and width implemented are the optimal ones that result in maximum biomass accumulation efficiency, as found by optimization modeling using the 2D branch extension formulation.

© 2021 The Authors
