# Supplementary figures and images for: Collective colony growth is optimized by branching pattern formation in Pseudomonas aeruginosa
Source: Mol Syst Biol. 2021 Apr 26;17(4):e10089. doi: 10.15252/msb.202010089 (PMC8073002; doi:10.15252/msb.202010089)

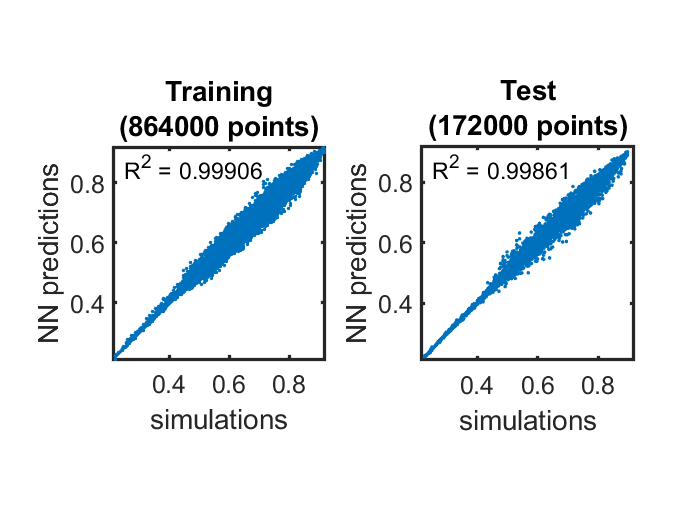

Supplement: Supplementary file 3 — Source Data for Expanded View and Appendix [file MSB-17-e10089-s003.zip › Source Data for Expanded View and Appendix/Appendix Figure S4_Neural networks for biomass prediction/NN1/correlation_pred.png]

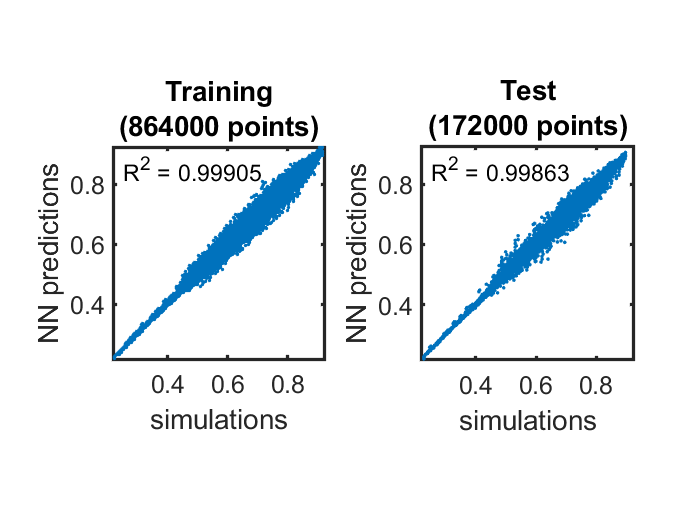

Supplement: Supplementary file 3 — Source Data for Expanded View and Appendix [file MSB-17-e10089-s003.zip › Source Data for Expanded View and Appendix/Appendix Figure S4_Neural networks for biomass prediction/NN2/correlation_pred.png]

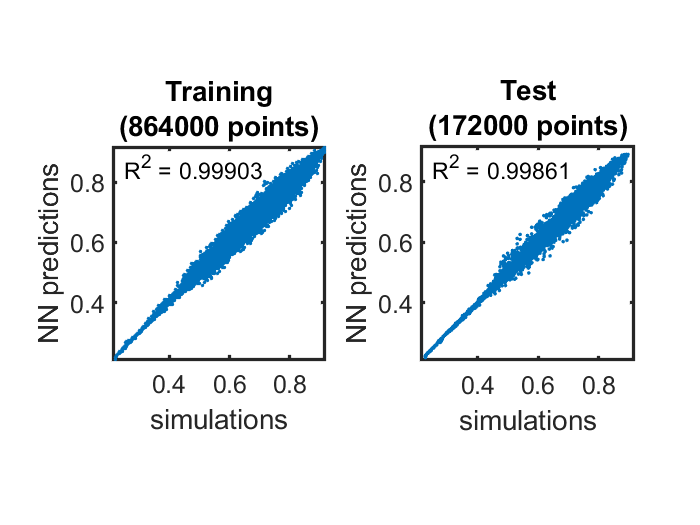

Supplement: Supplementary file 3 — Source Data for Expanded View and Appendix [file MSB-17-e10089-s003.zip › Source Data for Expanded View and Appendix/Appendix Figure S4_Neural networks for biomass prediction/NN3/correlation_pred.png]

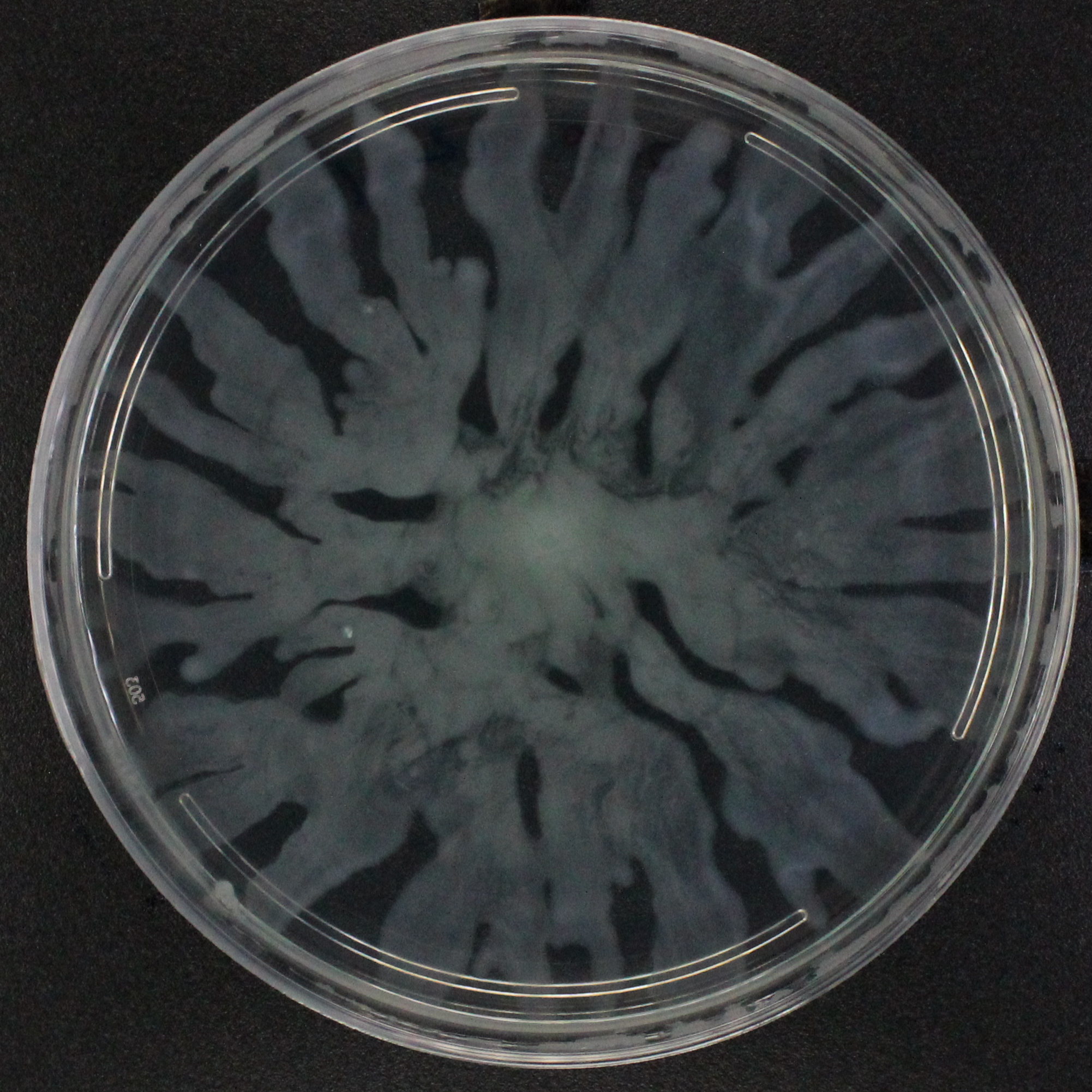

Supplement: Supplementary file 3 — Source Data for Expanded View and Appendix [file MSB-17-e10089-s003.zip › Source Data for Expanded View and Appendix/Figure EV2/Measure branches/sample images/12-0.40-1.TIF]

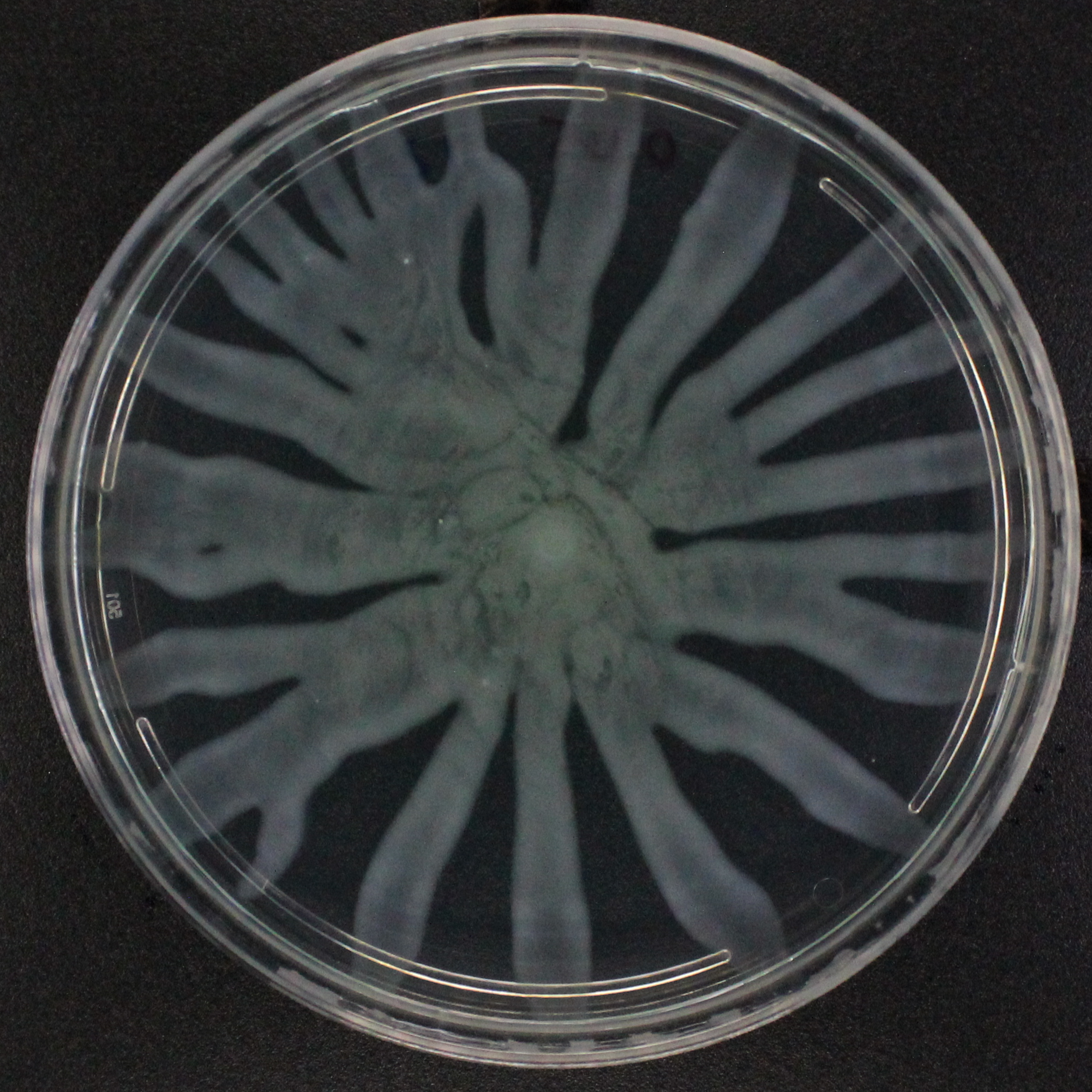

Supplement: Supplementary file 3 — Source Data for Expanded View and Appendix [file MSB-17-e10089-s003.zip › Source Data for Expanded View and Appendix/Figure EV2/Measure branches/sample images/12-0.45-1.TIF]

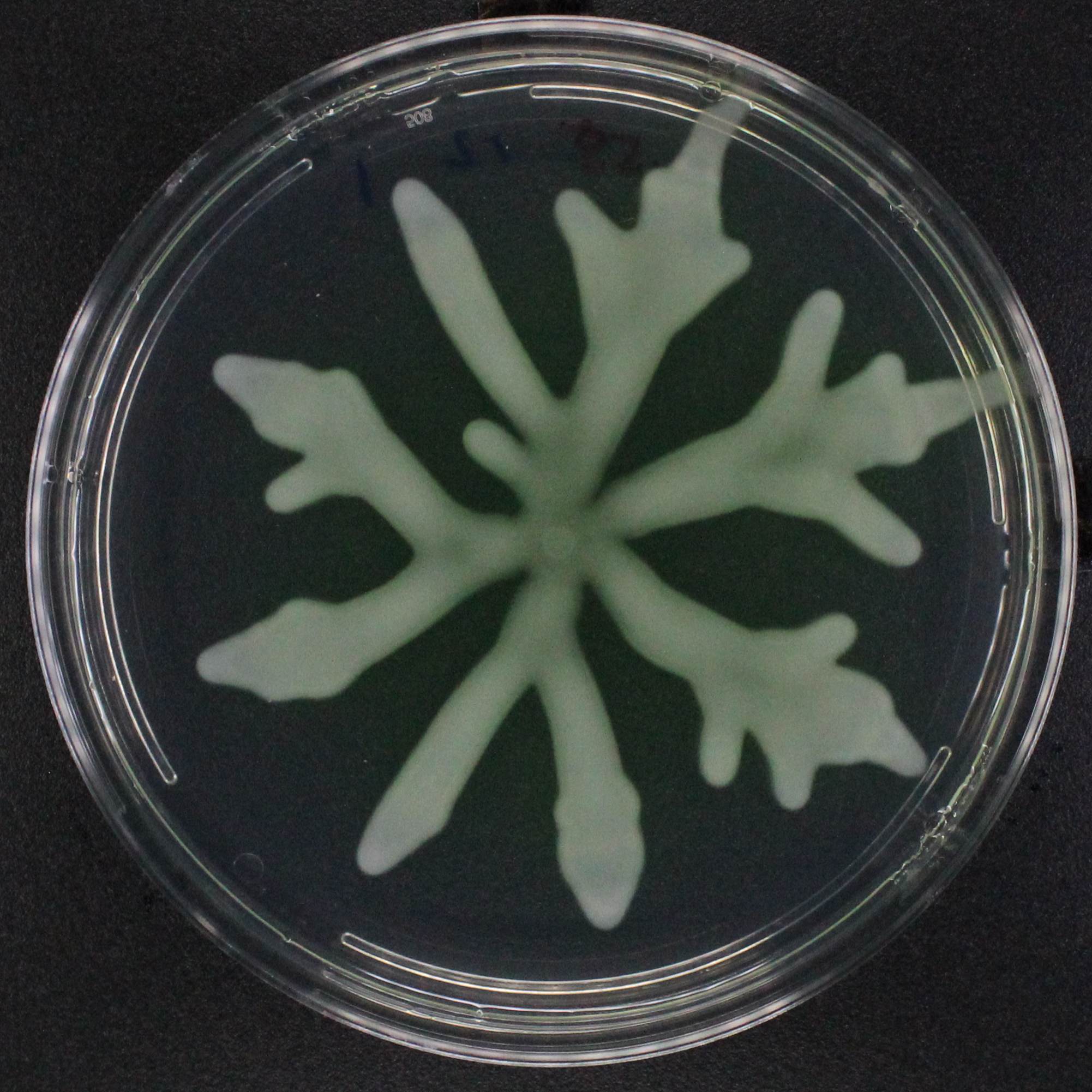

Supplement: Supplementary file 3 — Source Data for Expanded View and Appendix [file MSB-17-e10089-s003.zip › Source Data for Expanded View and Appendix/Figure EV2/Measure branches/sample images/12-0.55-1.TIF]

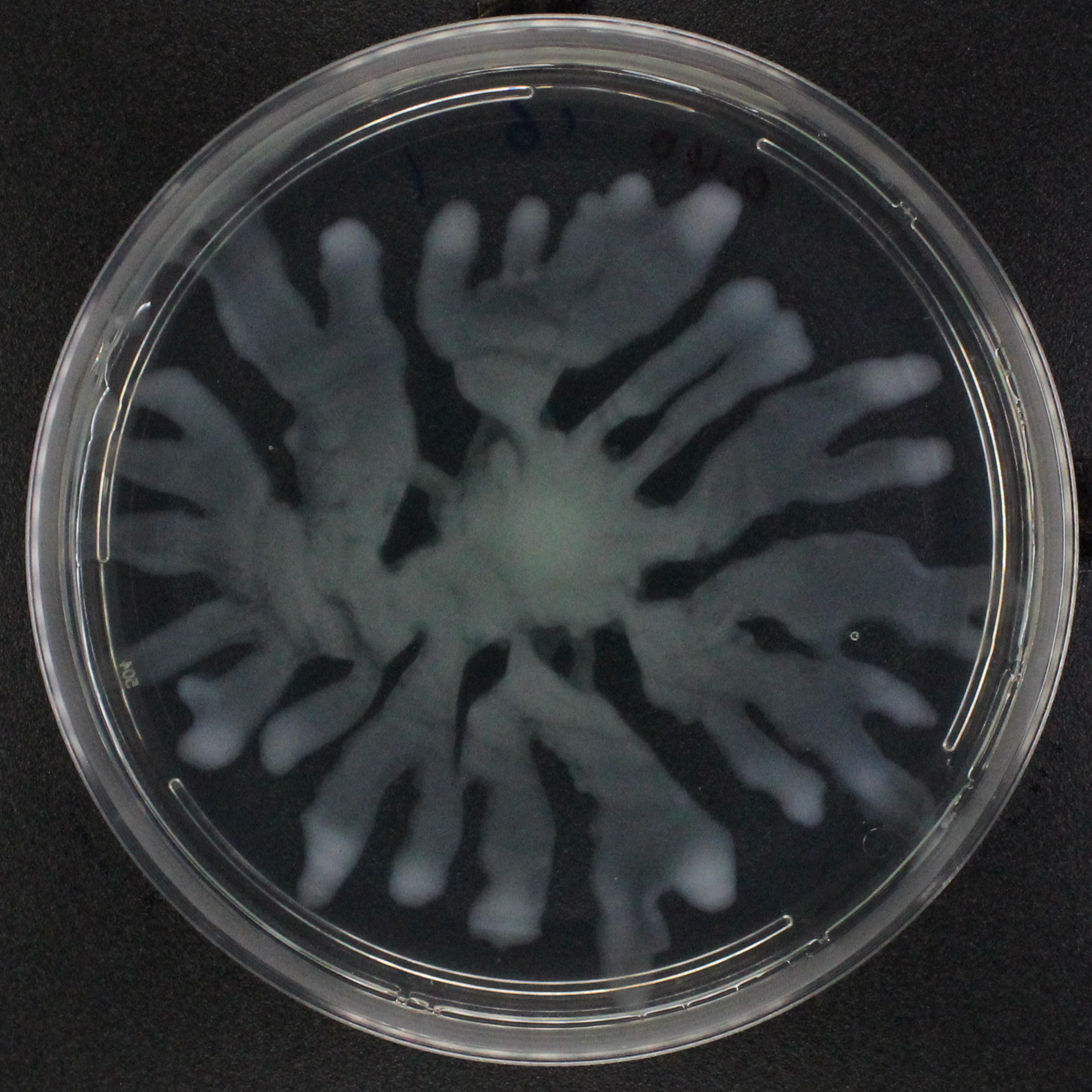

Supplement: Supplementary file 3 — Source Data for Expanded View and Appendix [file MSB-17-e10089-s003.zip › Source Data for Expanded View and Appendix/Figure EV2/Measure branches/sample images/16-0.40-1.TIF]

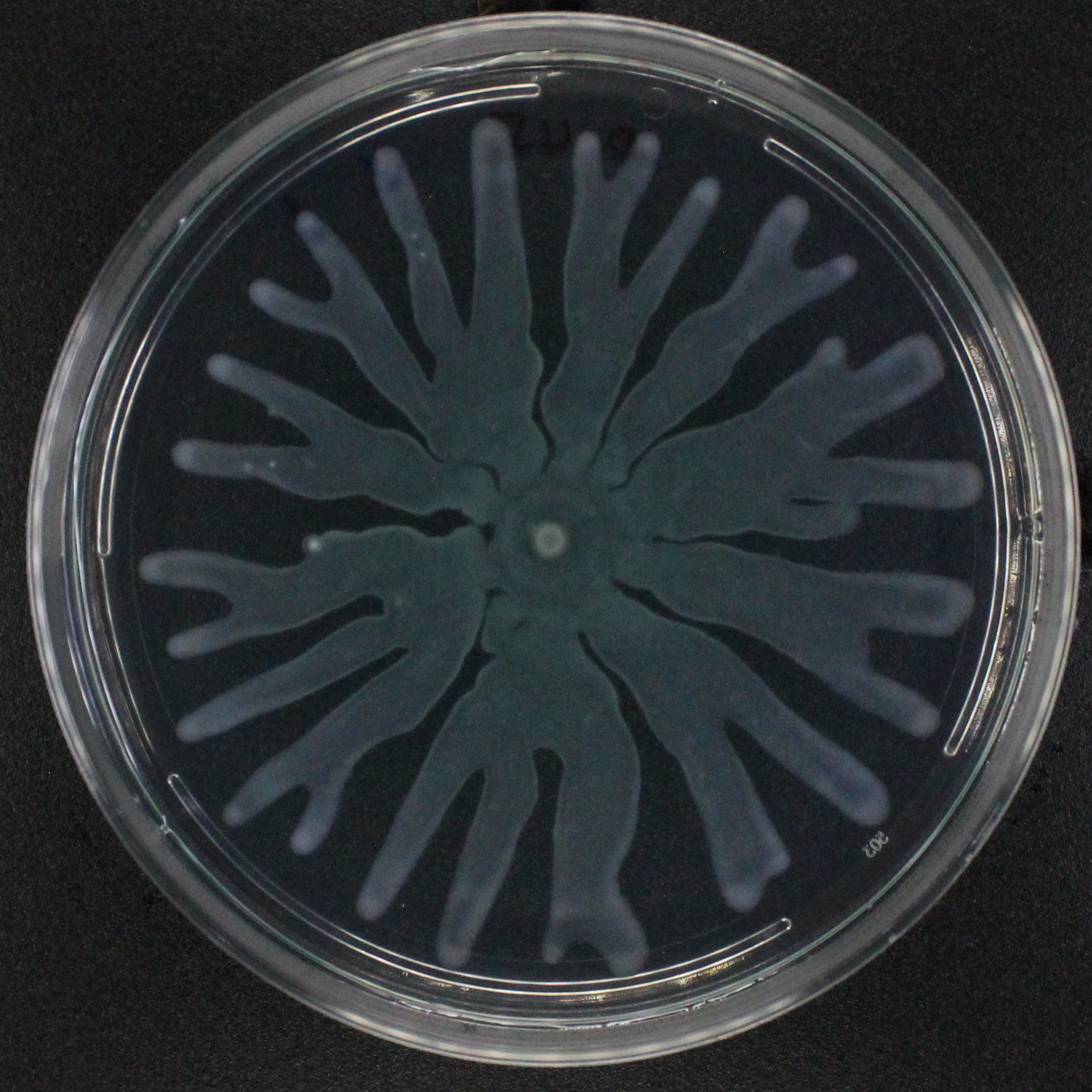

Supplement: Supplementary file 3 — Source Data for Expanded View and Appendix [file MSB-17-e10089-s003.zip › Source Data for Expanded View and Appendix/Figure EV2/Measure branches/sample images/4-0.45-1.TIF]

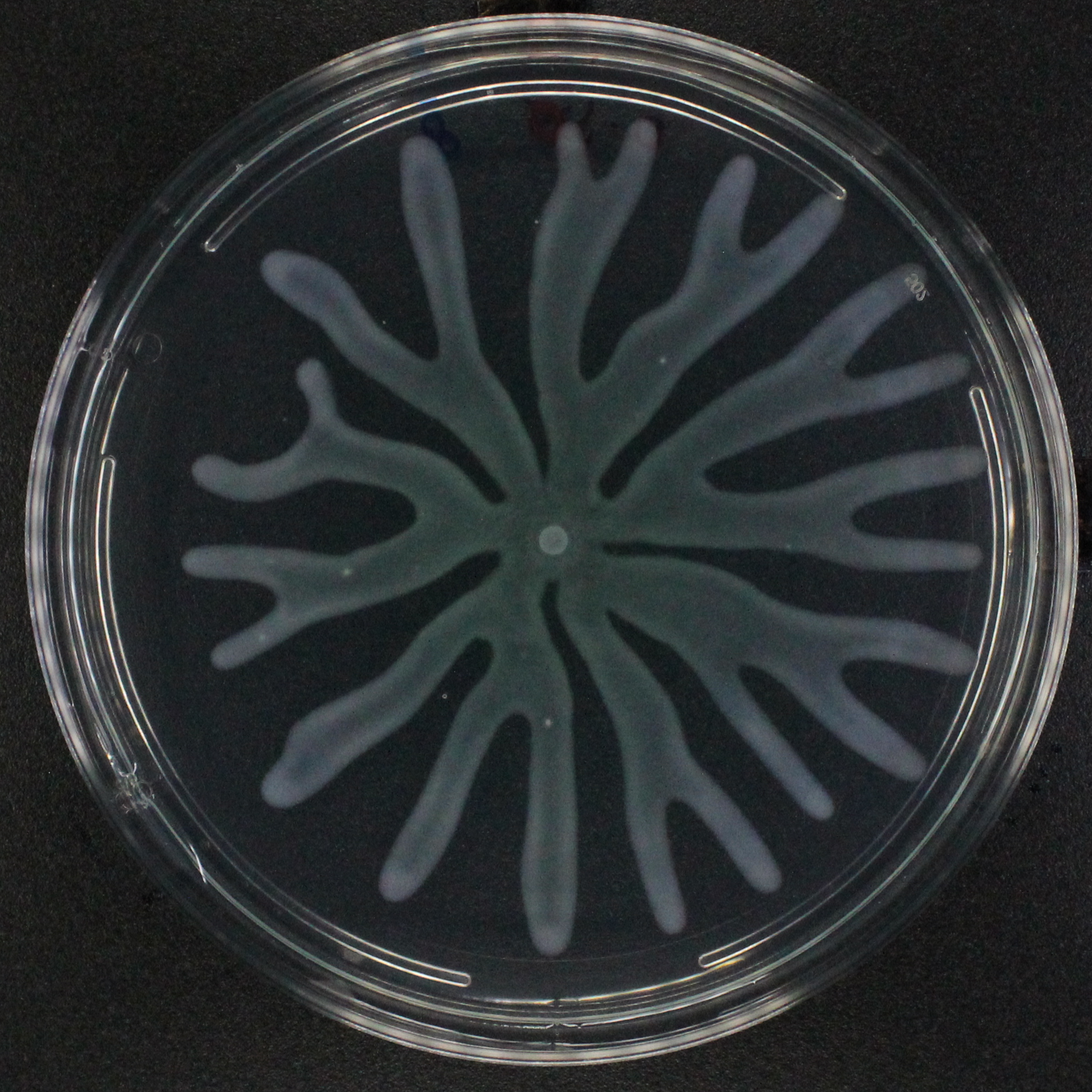

Supplement: Supplementary file 3 — Source Data for Expanded View and Appendix [file MSB-17-e10089-s003.zip › Source Data for Expanded View and Appendix/Figure EV2/Measure branches/sample images/8-0.50-1.TIF]

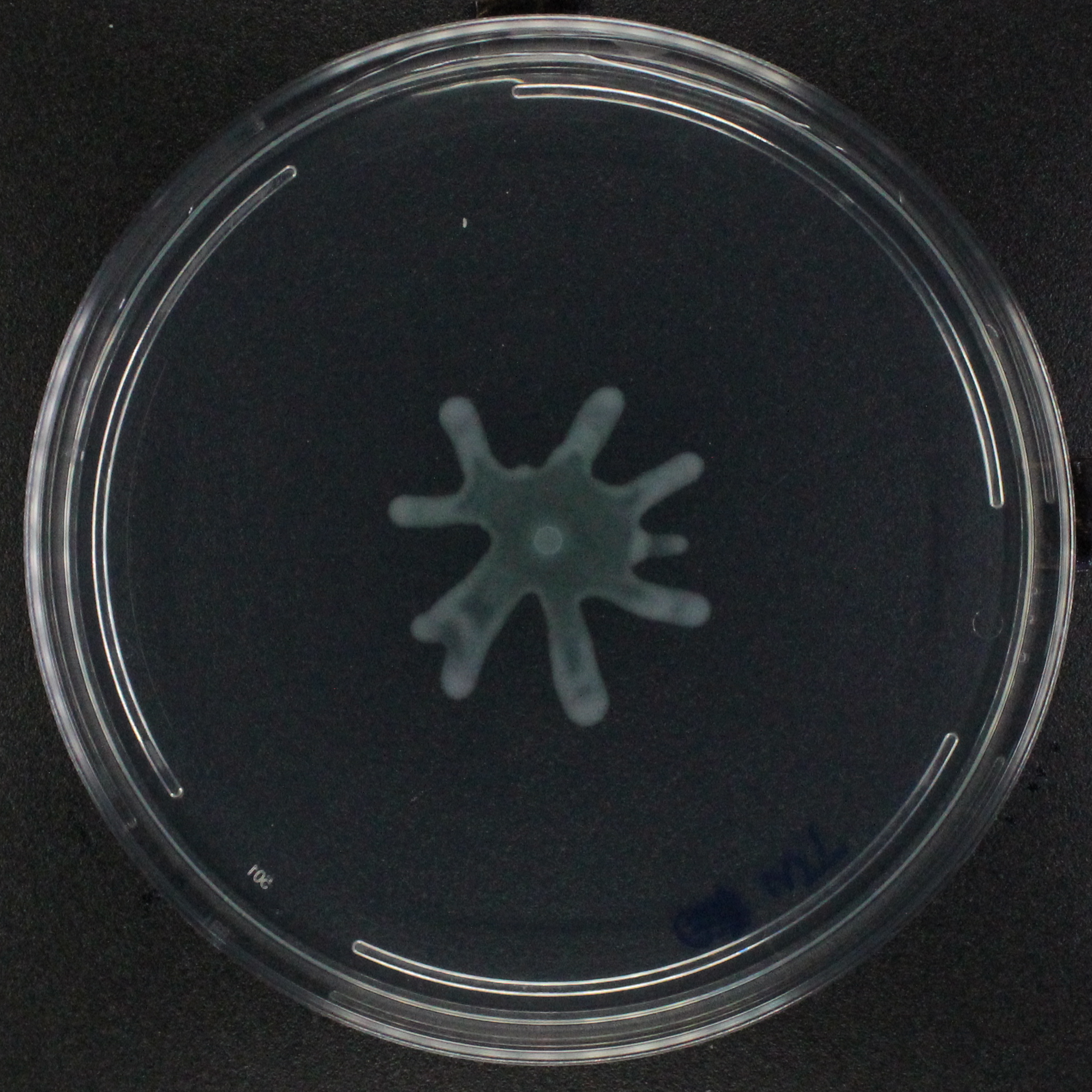

Supplement: Supplementary file 5 — Source Data for Figure 1 [file MSB-17-e10089-s005.zip › Source data for Figure 1/Figure 1A/12h_WT-3.TIF]

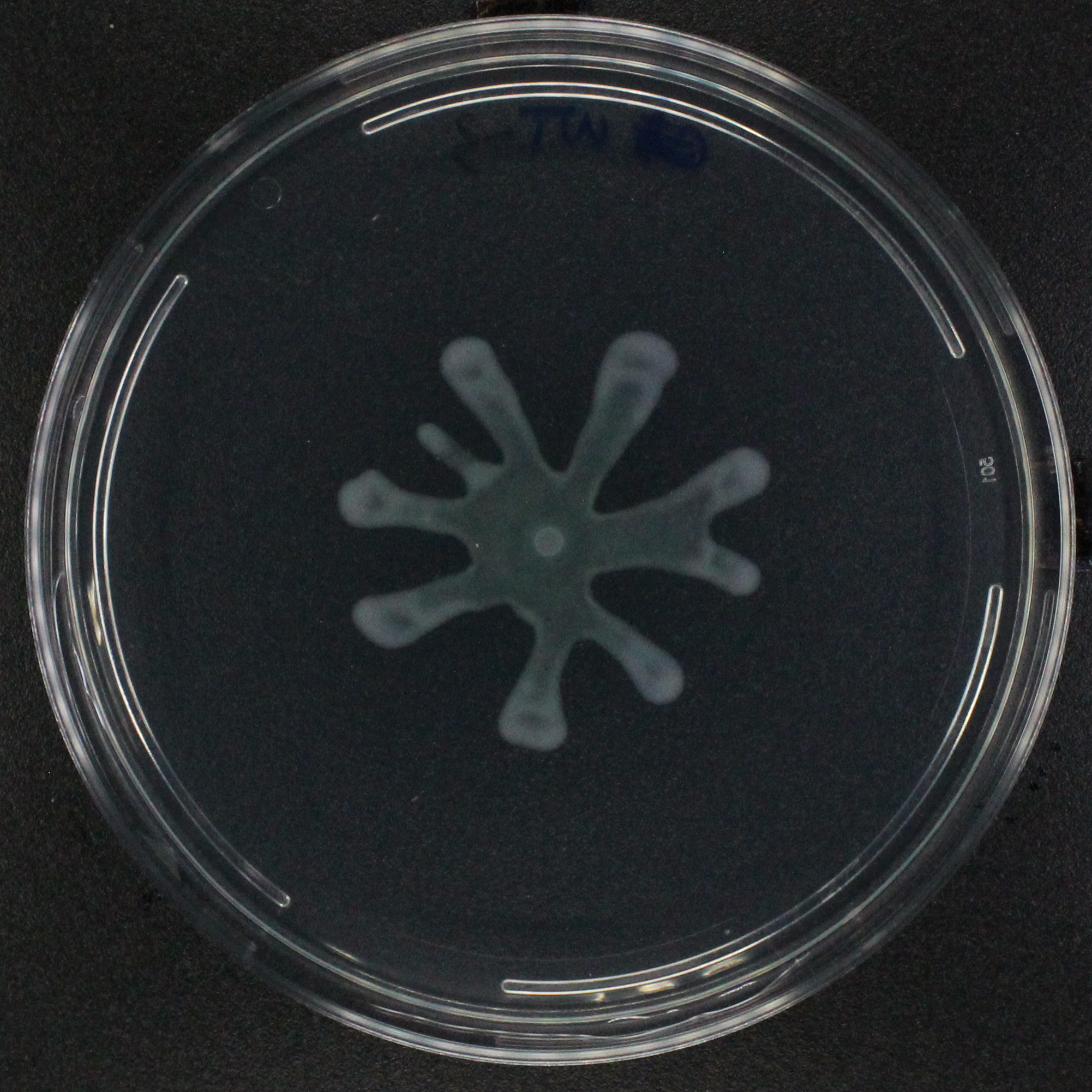

Supplement: Supplementary file 5 — Source Data for Figure 1 [file MSB-17-e10089-s005.zip › Source data for Figure 1/Figure 1A/13h_WT-3.TIF]

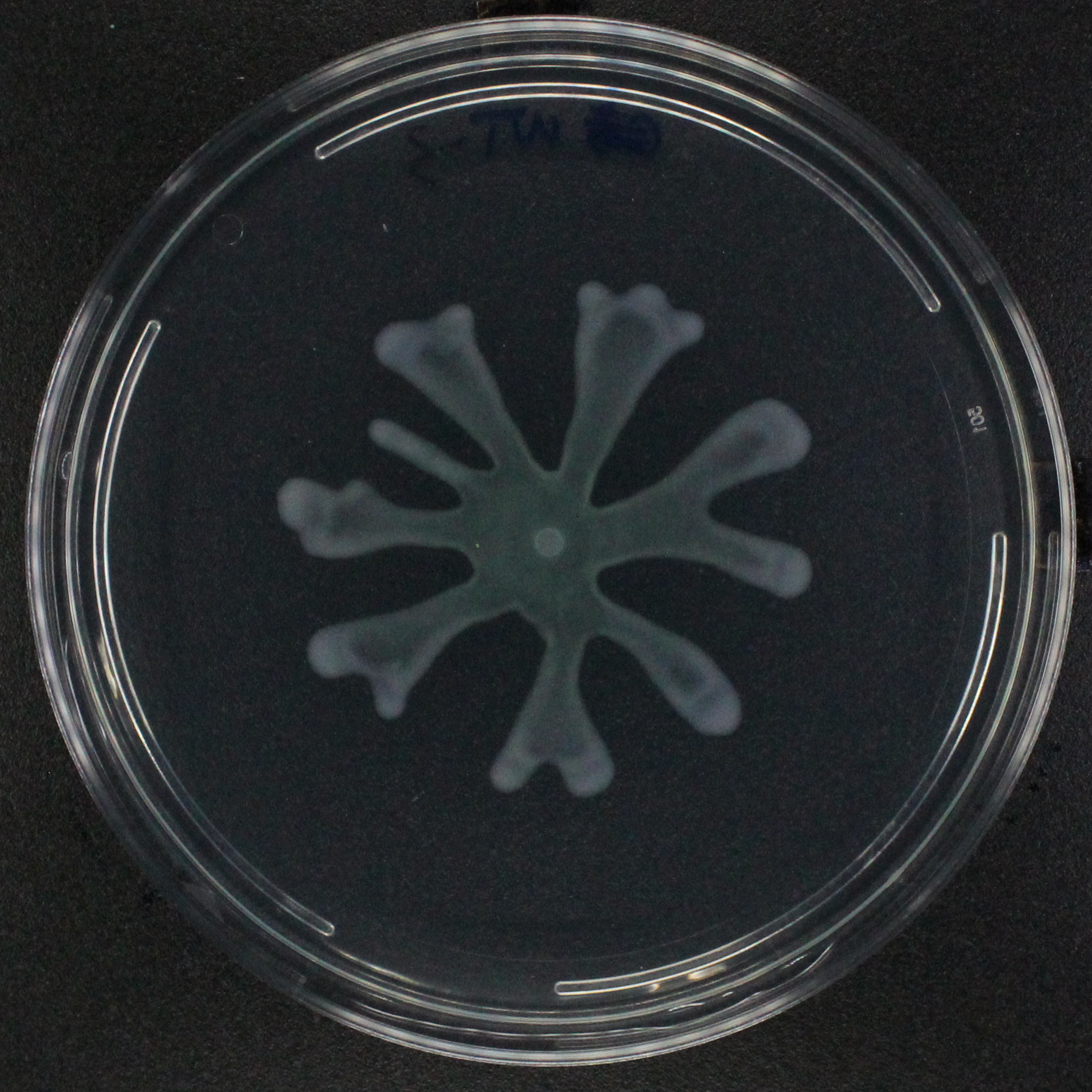

Supplement: Supplementary file 5 — Source Data for Figure 1 [file MSB-17-e10089-s005.zip › Source data for Figure 1/Figure 1A/14h_WT-3.TIF]

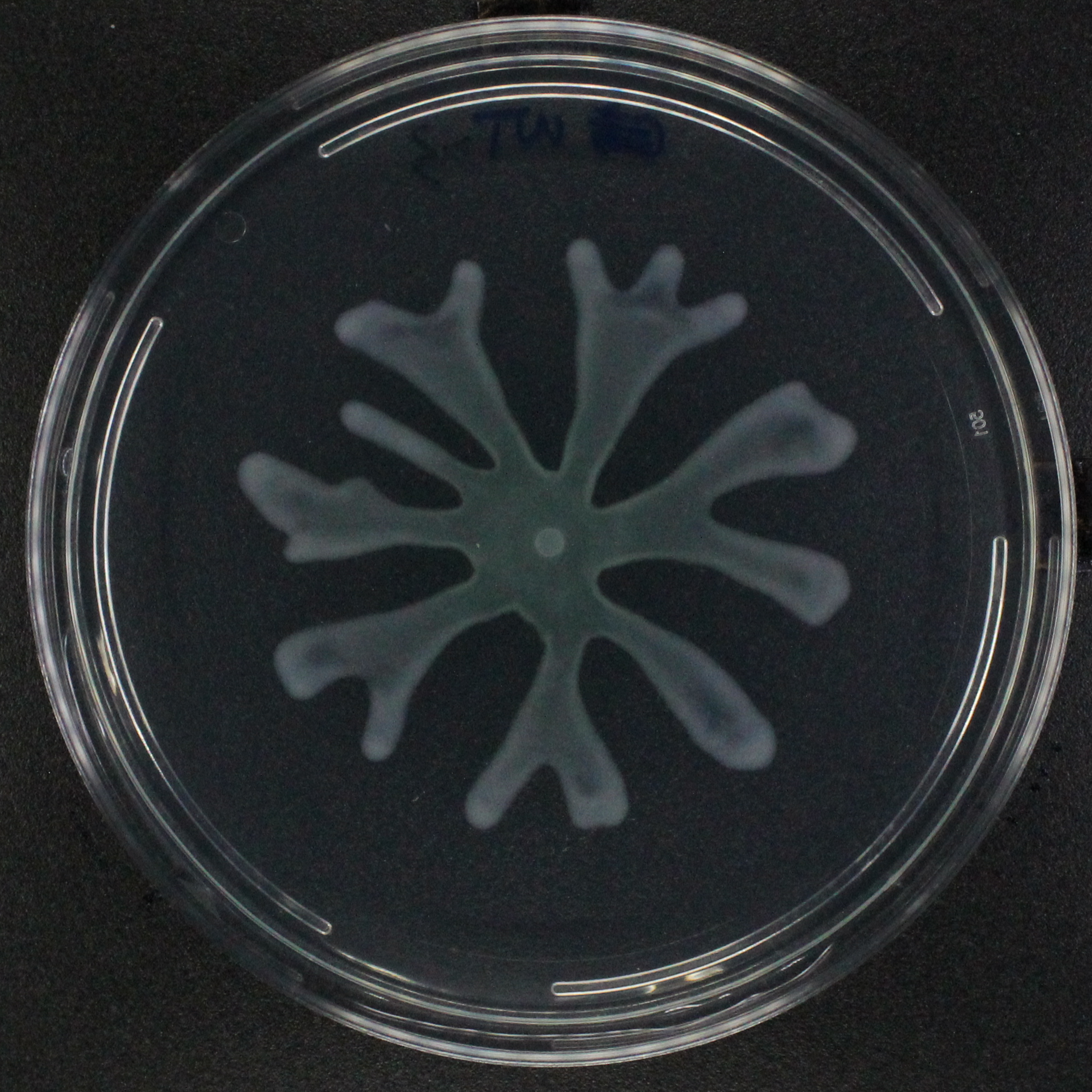

Supplement: Supplementary file 5 — Source Data for Figure 1 [file MSB-17-e10089-s005.zip › Source data for Figure 1/Figure 1A/15h_WT-3.TIF]

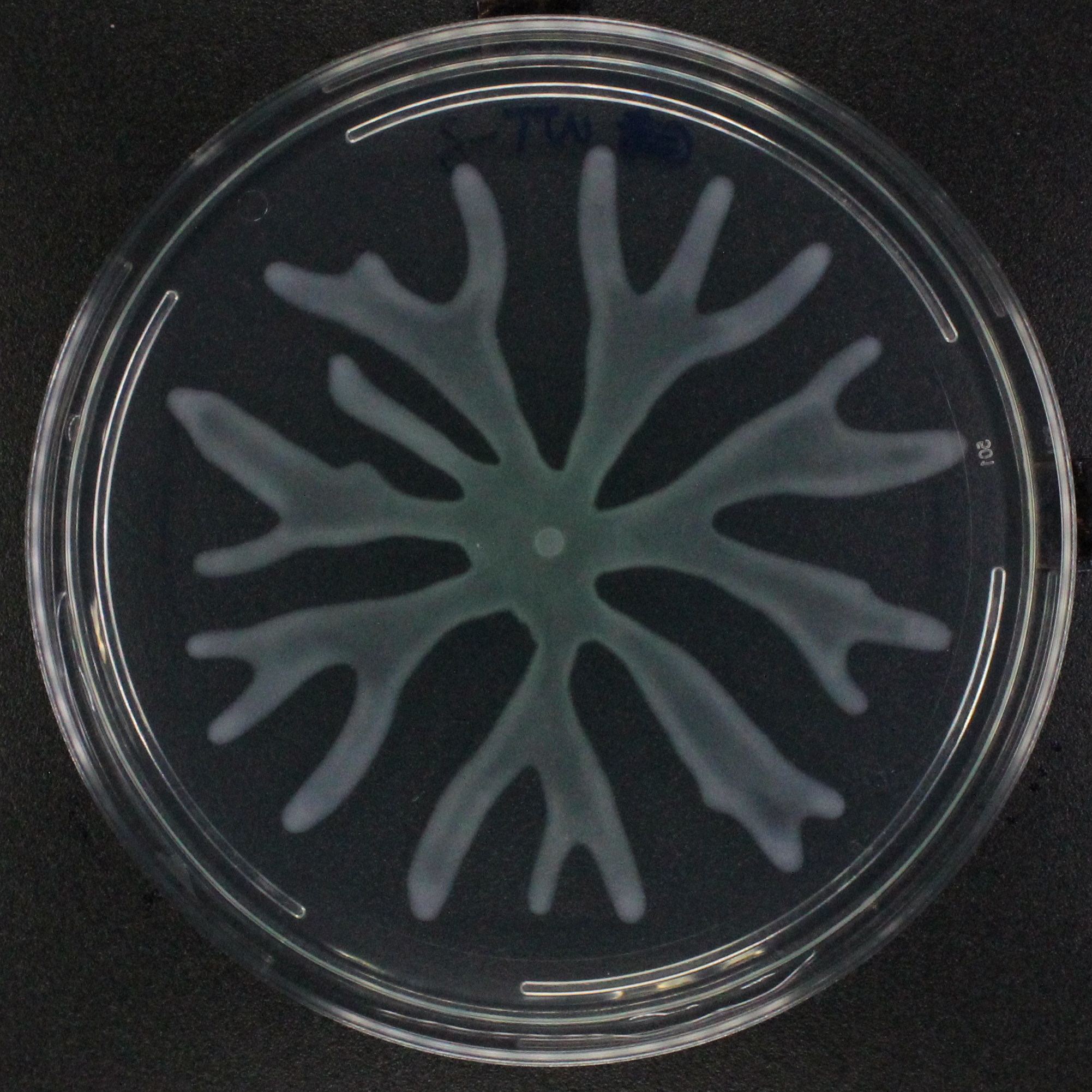

Supplement: Supplementary file 5 — Source Data for Figure 1 [file MSB-17-e10089-s005.zip › Source data for Figure 1/Figure 1A/17h_WT-3.TIF]

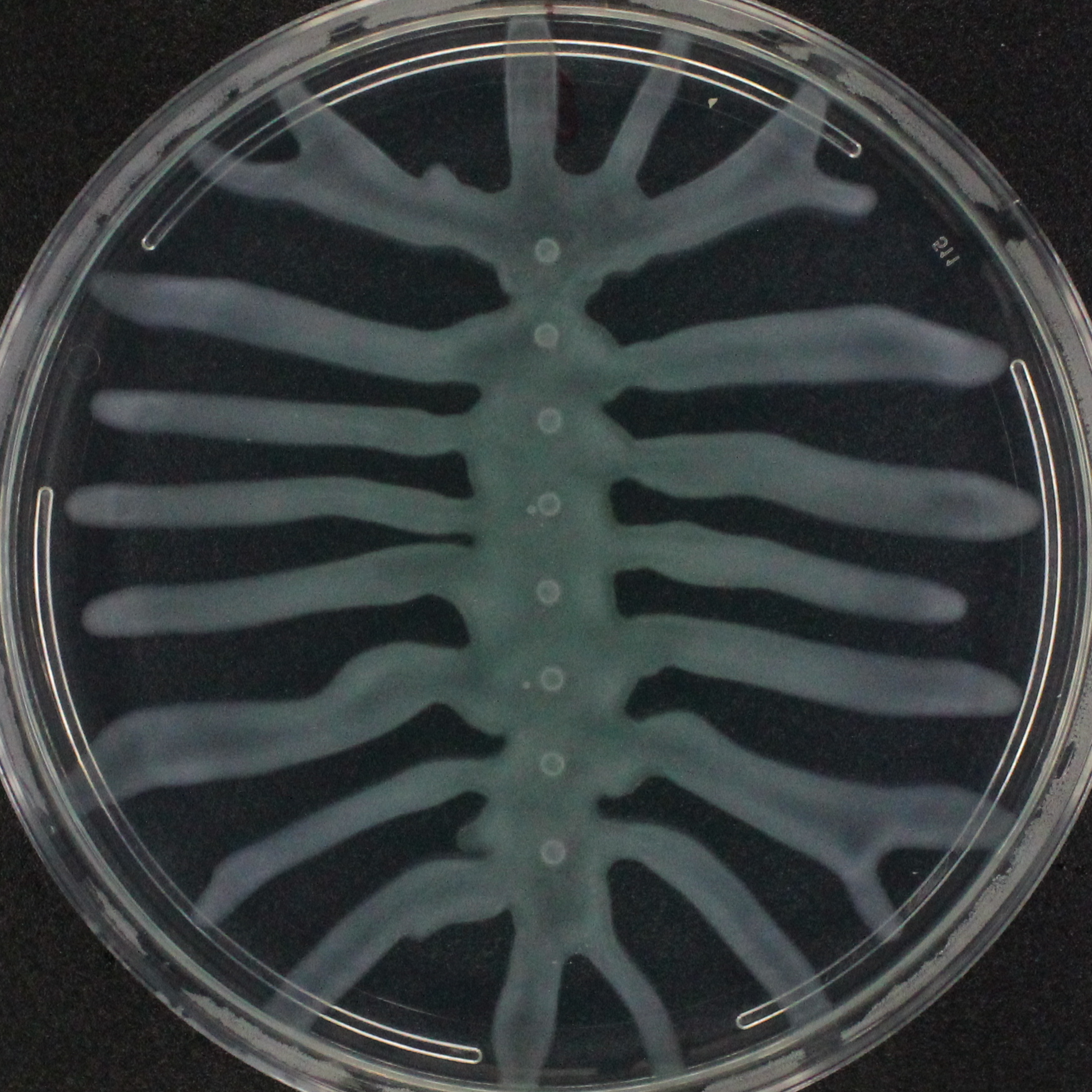

Supplement: Supplementary file 5 — Source Data for Figure 1 [file MSB-17-e10089-s005.zip › Source data for Figure 1/Figure 1B/Line inoculation.tif]

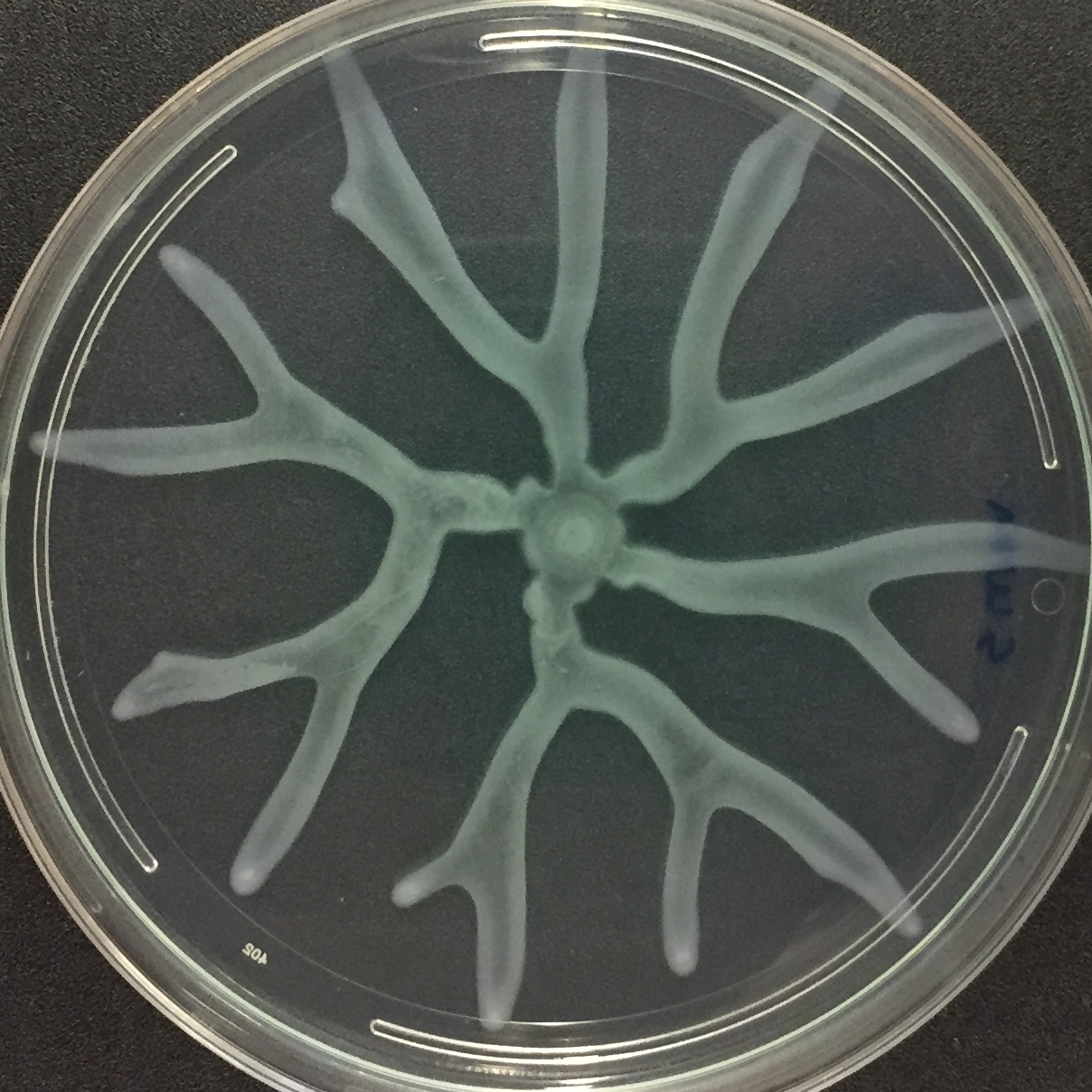

Supplement: Supplementary file 5 — Source Data for Figure 1 [file MSB-17-e10089-s005.zip › Source data for Figure 1/Figure 1B/Point inoculation.jpg]

0 10 0 10

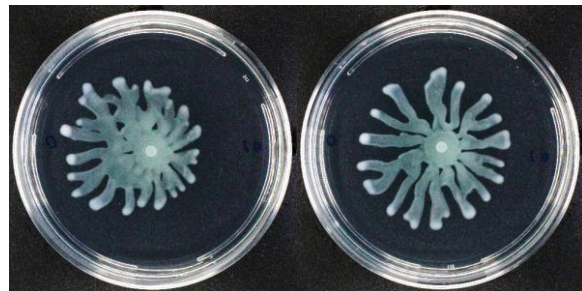

0 20 0 20

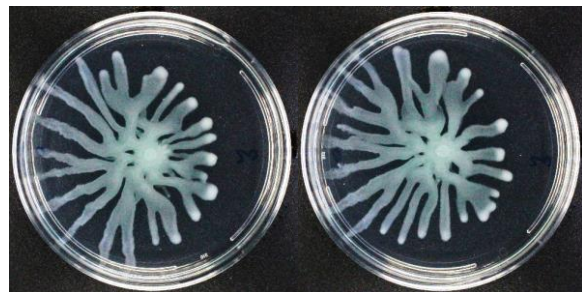

10

6

12

4

14

2

16

0

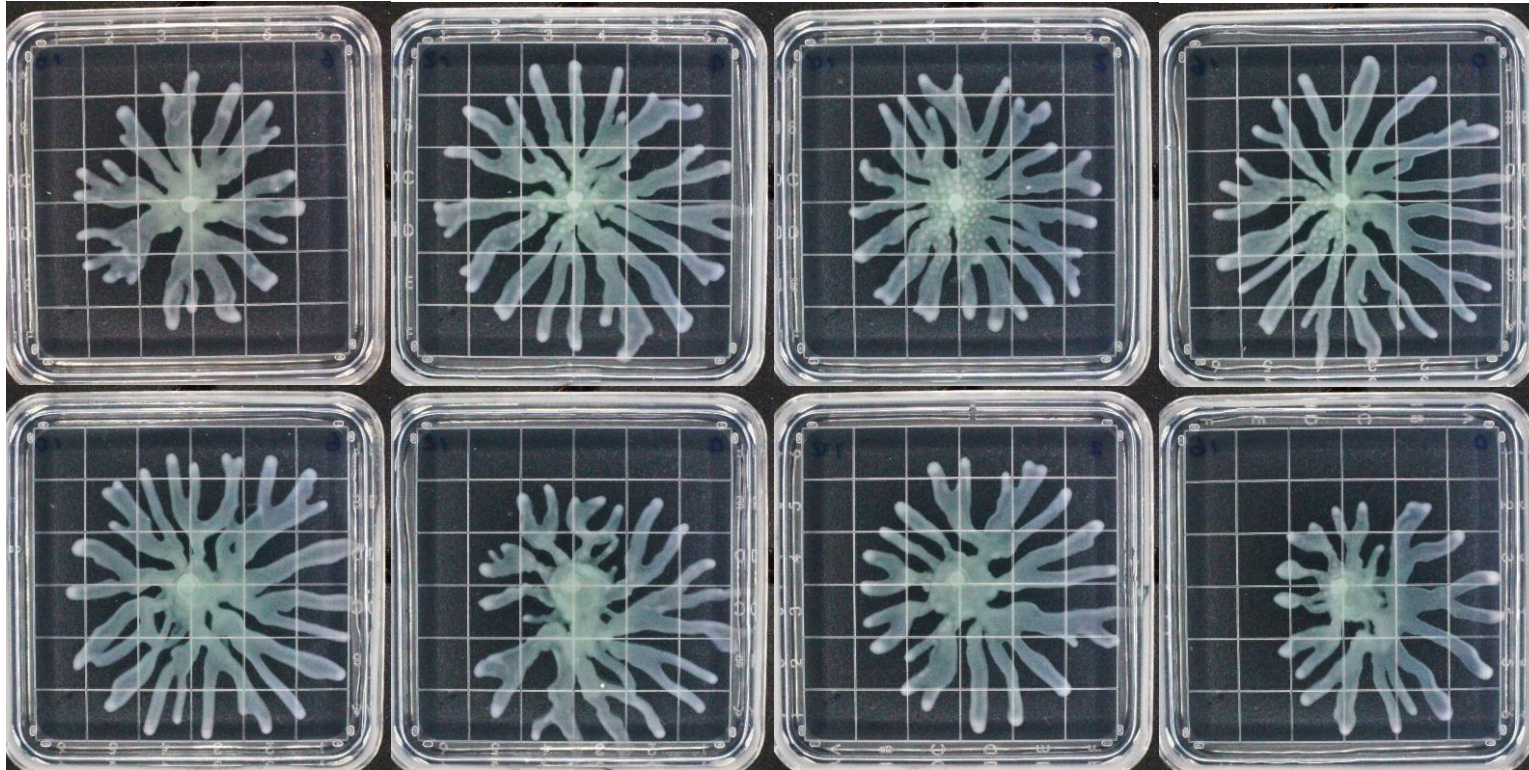

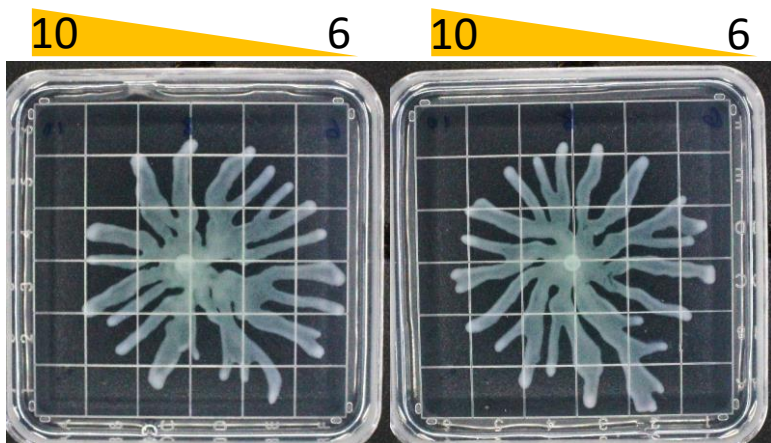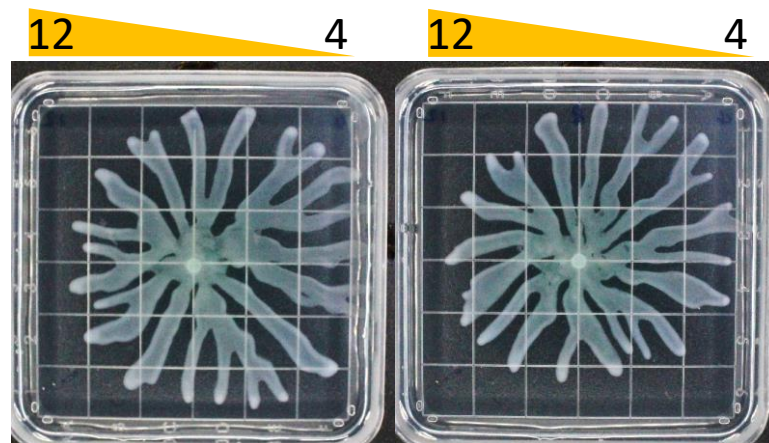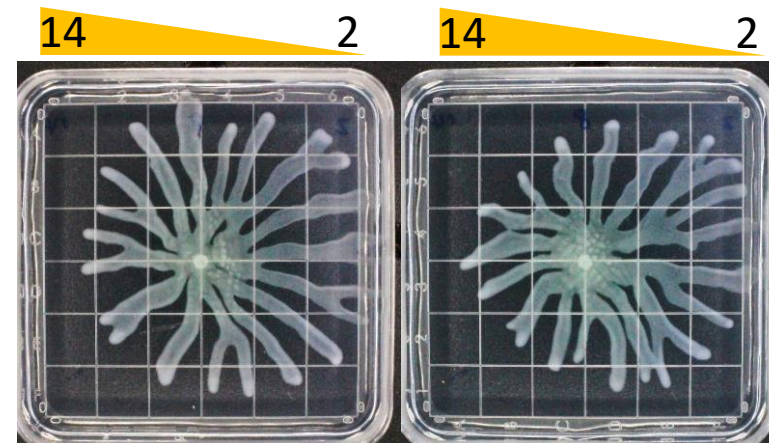

Supplement: Supplementary file 9 — Source Data for Figure 5 [file MSB-17-e10089-s006.zip › Source data for Figure 5/Figure 5 replicates.pdf]

4 g/L

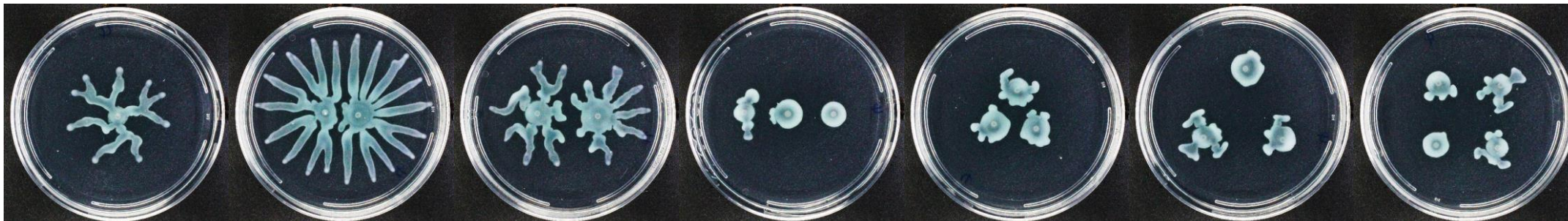

8 g/L

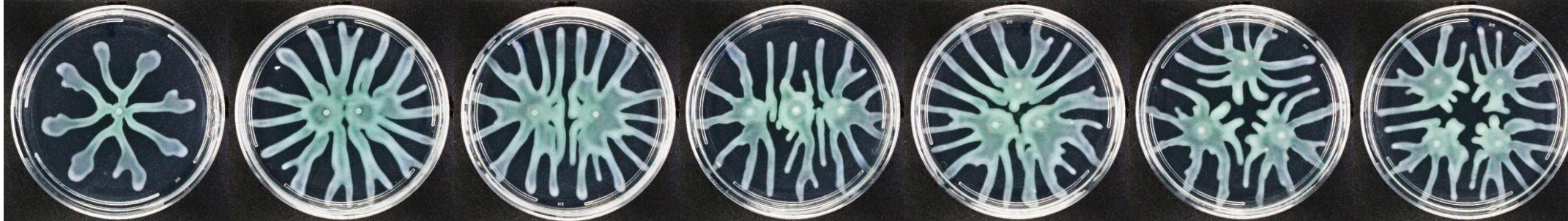

16 g/L

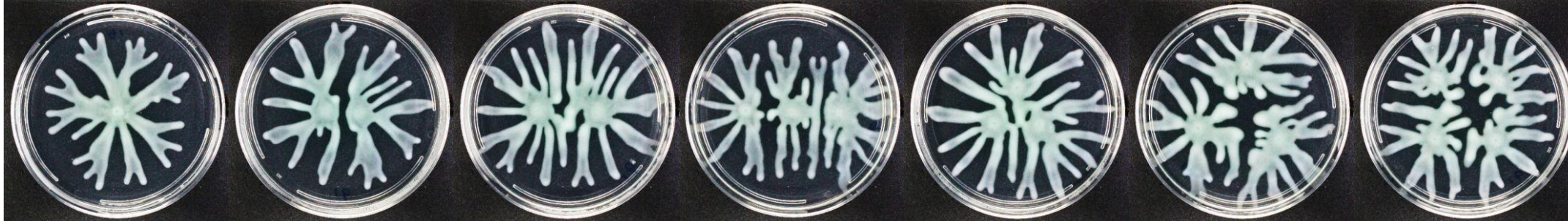

4 g/L

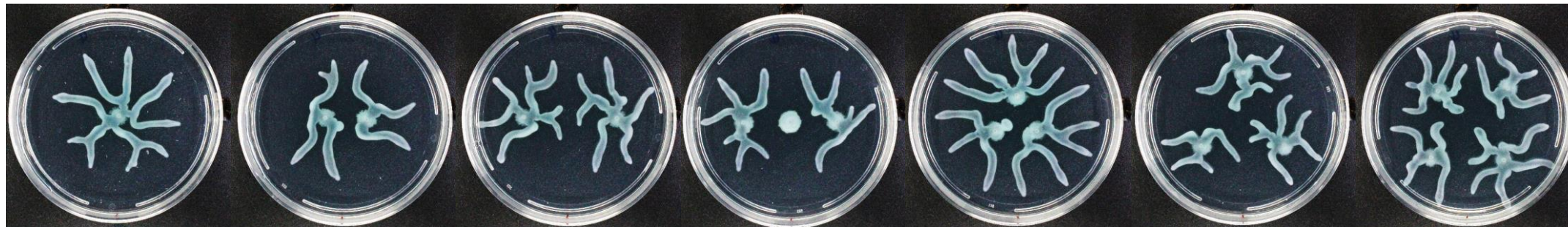

8 g/L

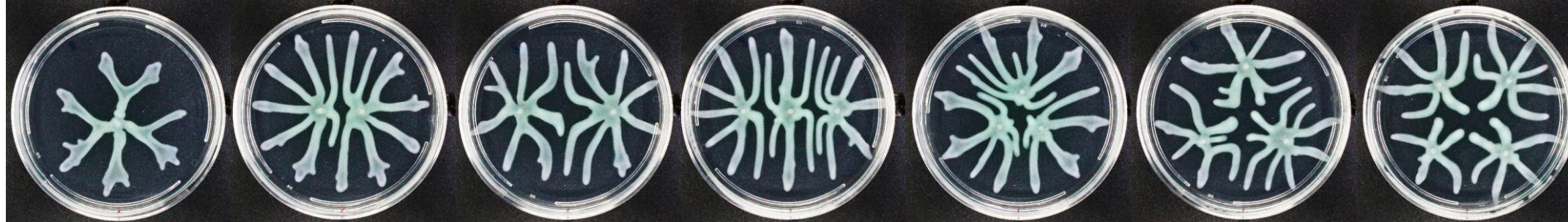

16 g/L

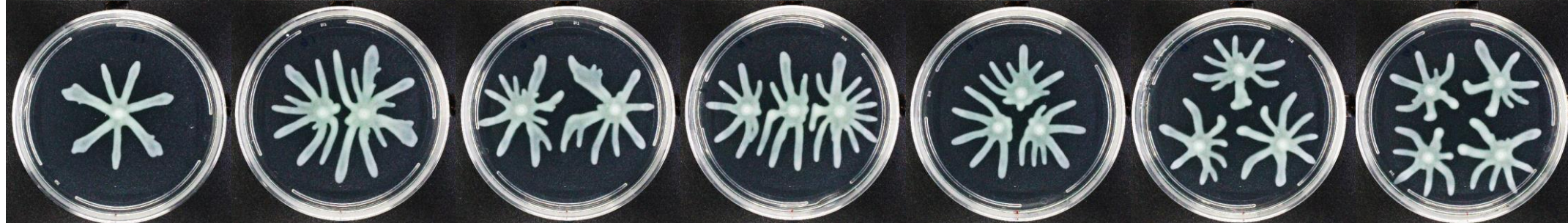

4

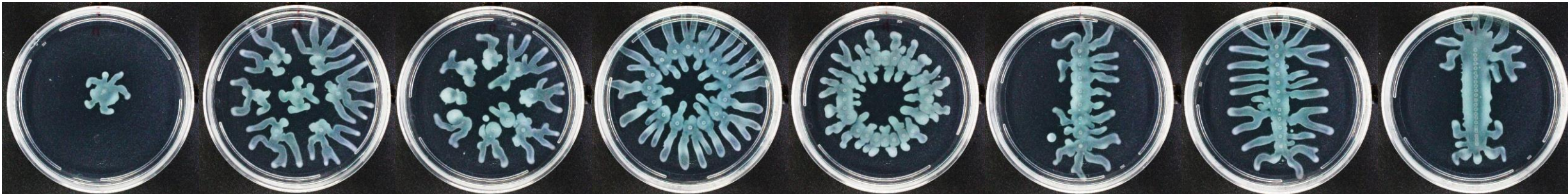

8

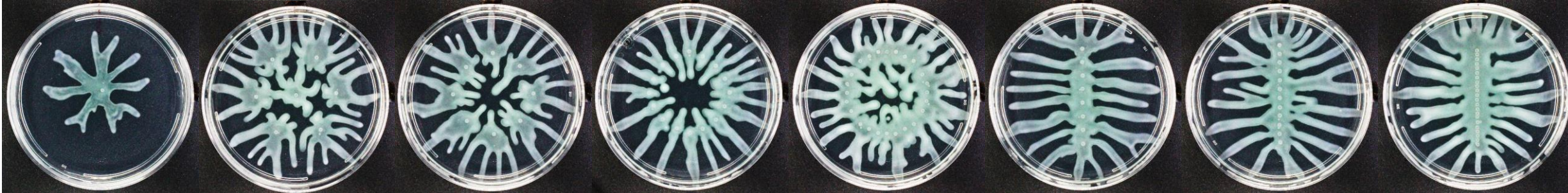

16

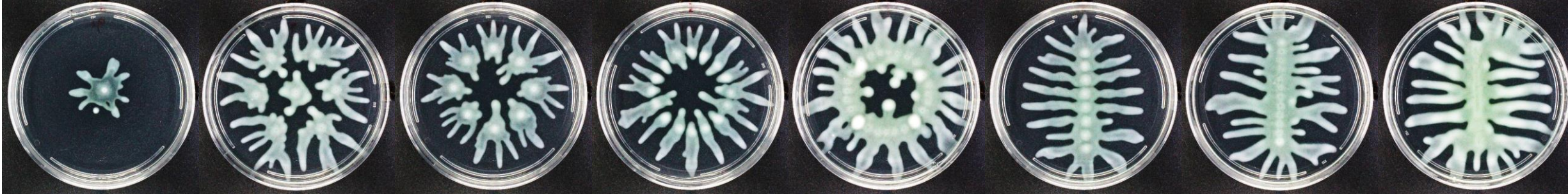

Supplement: Supplementary file 10 — Source Data for Figure 6 [file MSB-17-e10089-s007.zip › Source data for Figure 6/Figure 6 & EV5 replicates.pdf]
