# Supplementary material for: Collective colony growth is optimized by branching pattern formation in Pseudomonas aeruginosa
Source: Mol Syst Biol. 2021 Apr 26;17(4):e10089. doi: 10.15252/msb.202010089 (PMC8073002; doi:10.15252/msb.202010089)

Replicates of
Figure 2B panel a

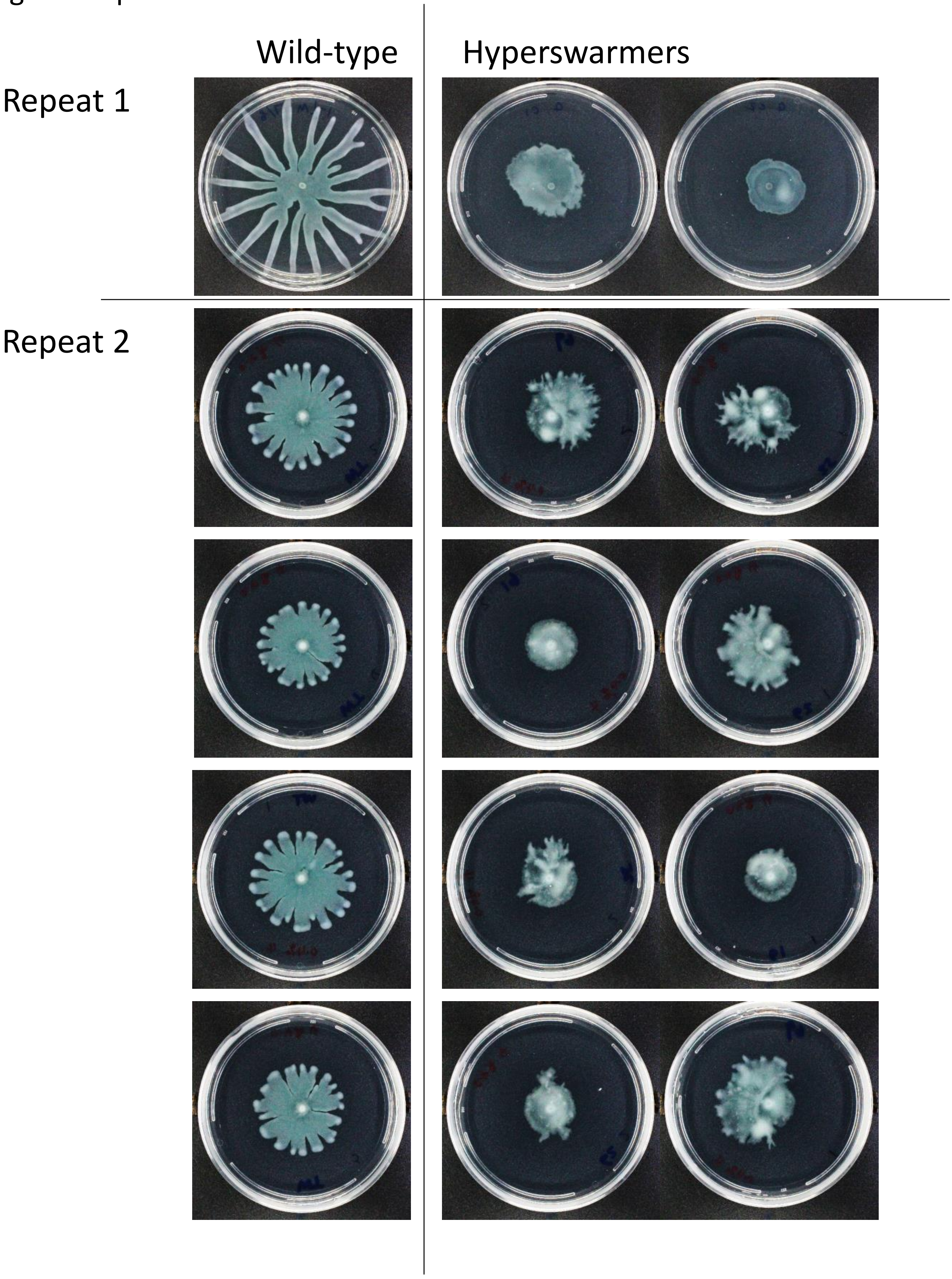

Replicates of
Figure 2B panel b

Wild-type | Hyperswarmers

Repeat 1

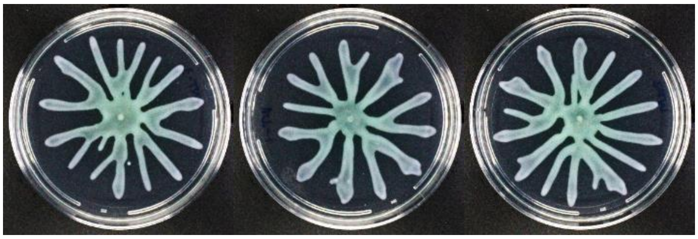

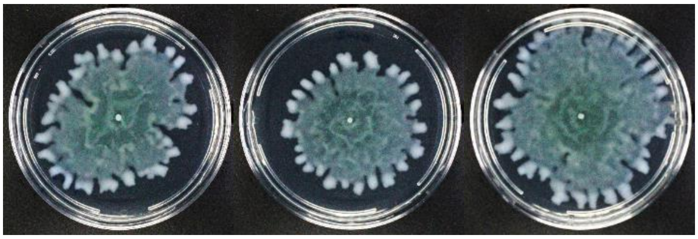

Repeat 2

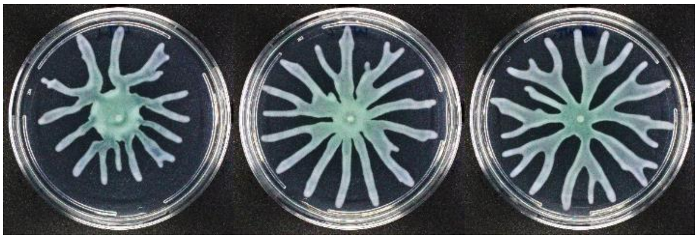

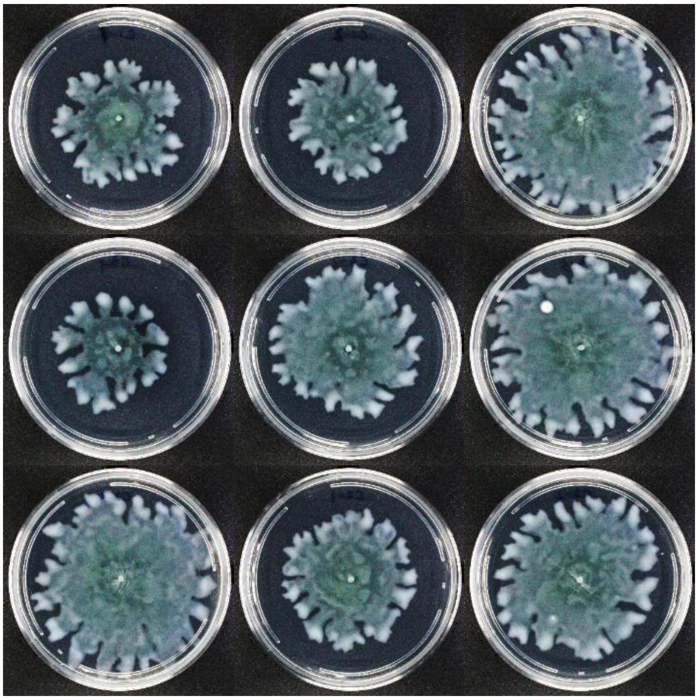

Replicates of
Figure 2B panel b

Wild-type | Hyperswarmers

Repeat 3

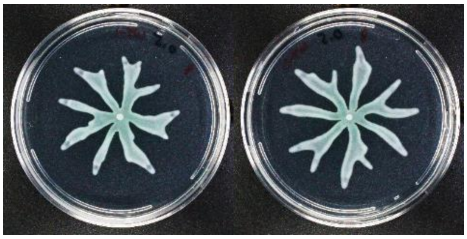

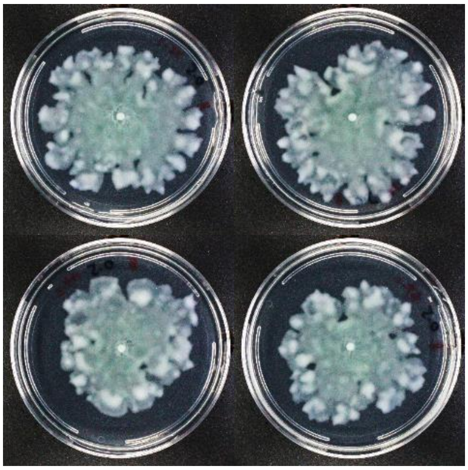

Repeat 4

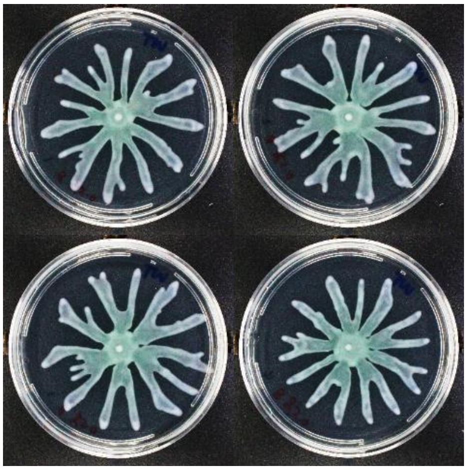

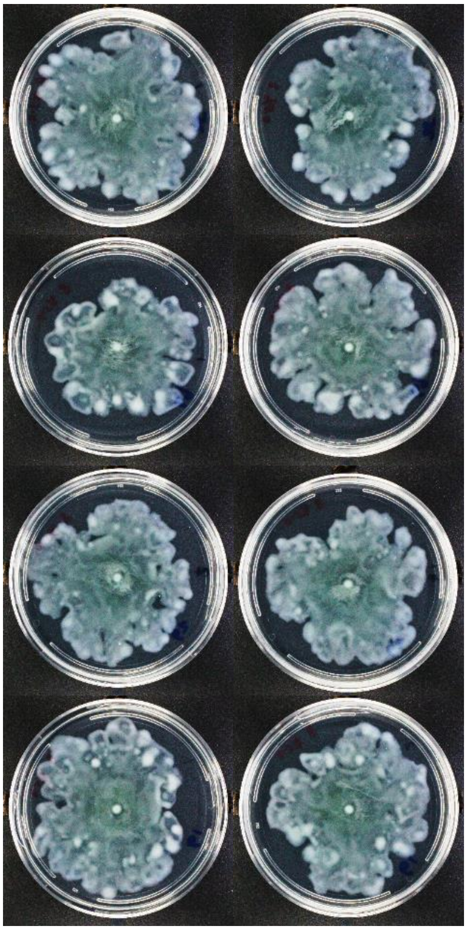

Replicates of
Figure 2B panel c

Wild-type | Hyperswarmers

Repeat 1

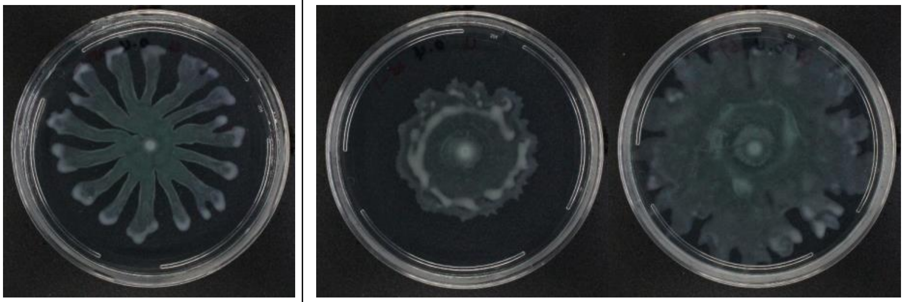

Repeat 2

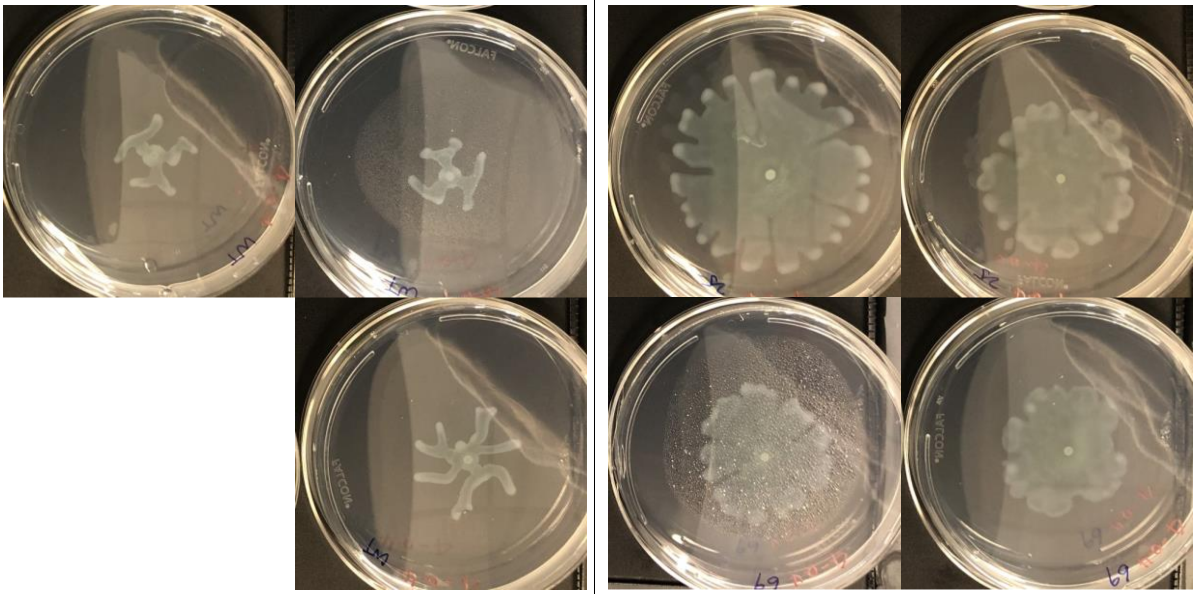

Supplement: Supplementary file 6 — Source Data for Figure 2 [file MSB-17-e10089-s002.zip › Source data for Figure 2/Figure 2 replicates.pdf]
