# Supplementary material for: Collective colony growth is optimized by branching pattern formation in Pseudomonas aeruginosa
Source: Mol Syst Biol. 2021 Apr 26;17(4):e10089. doi: 10.15252/msb.202010089 (PMC8073002; doi:10.15252/msb.202010089)

Replicate 1

Casamino acid

4 g/L 8 g/L 12 g/L 16 g/L

Agar density

0.55%

0.50%

0.45%

0.40%

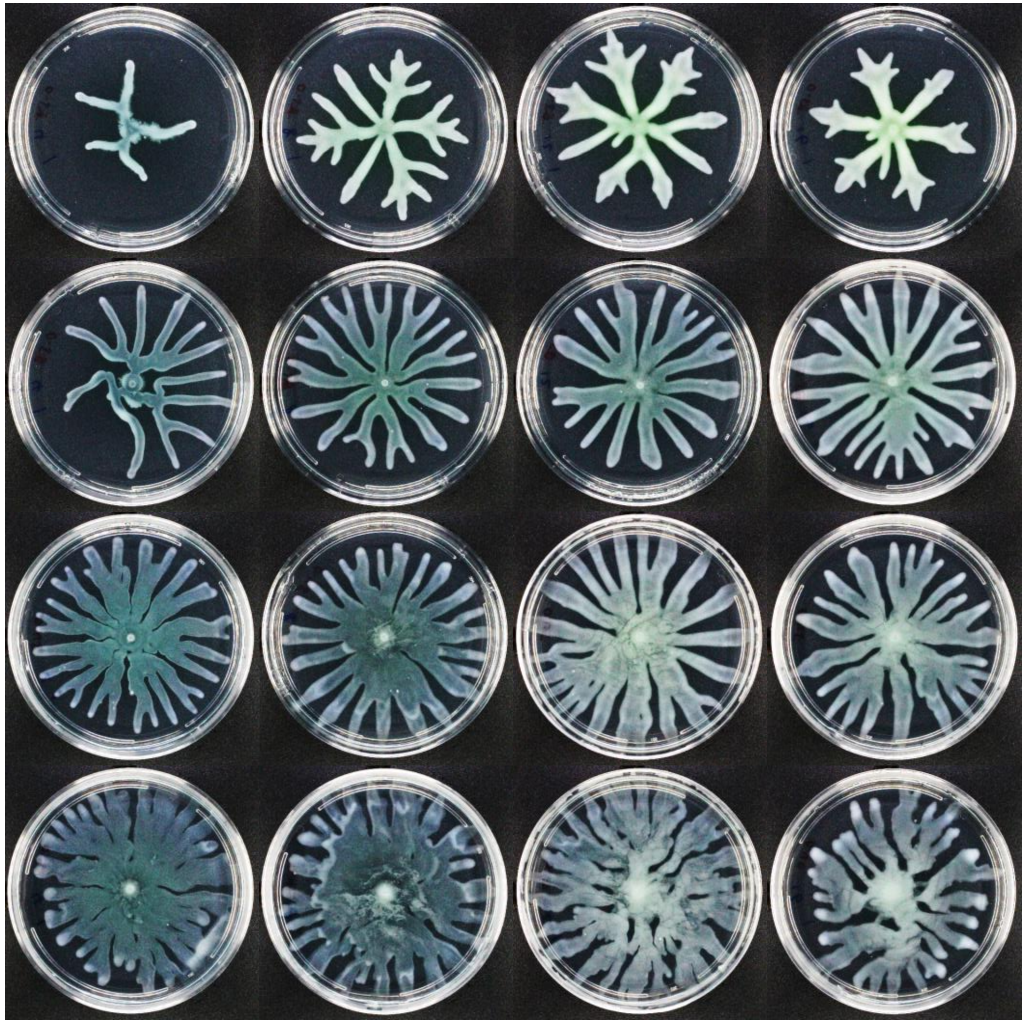

Replicate 2

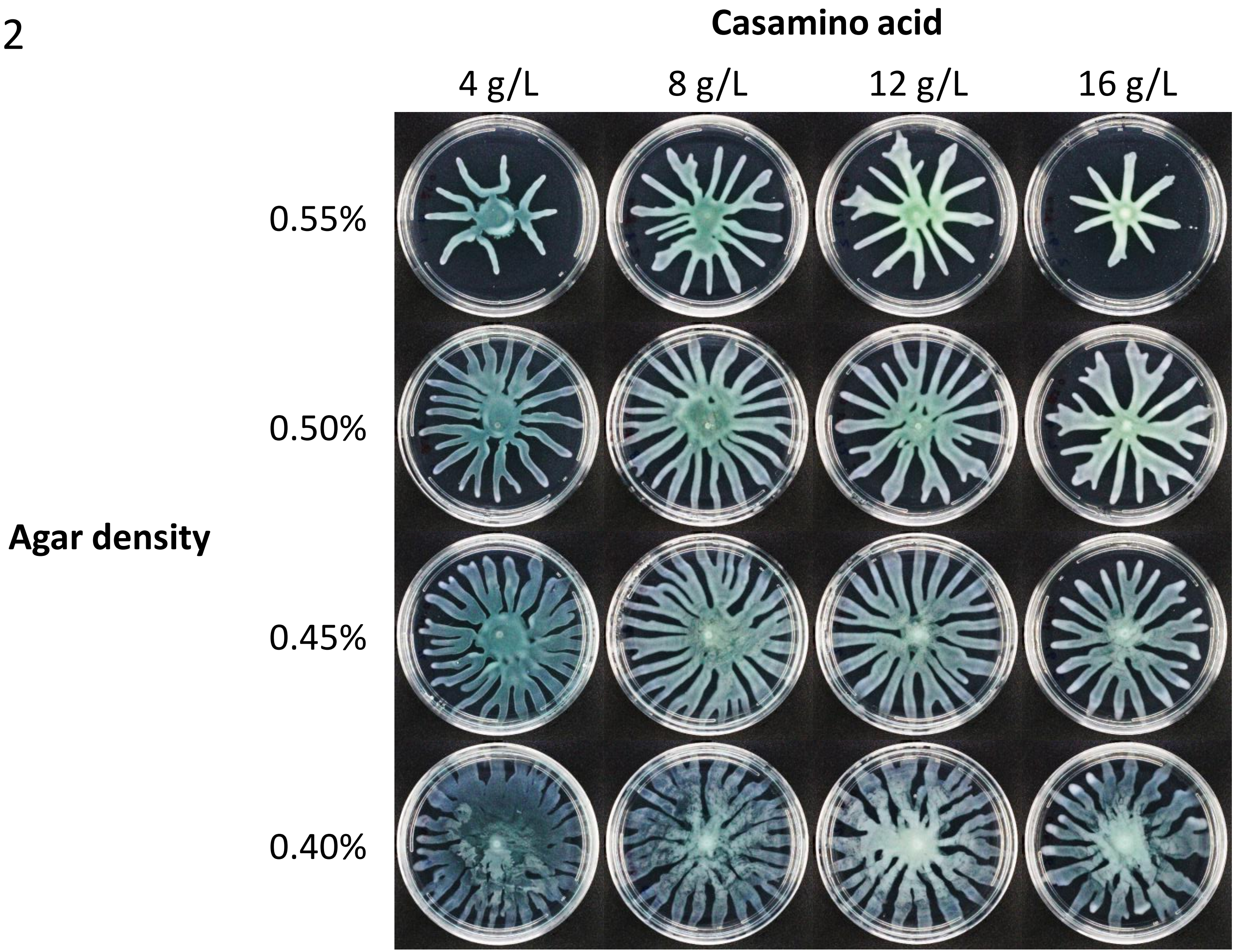

Replicate 3

Casamino acid

4 g/L 8 g/L 12 g/L

Agar density

0.55%

0.50%

0.45%

0.40%

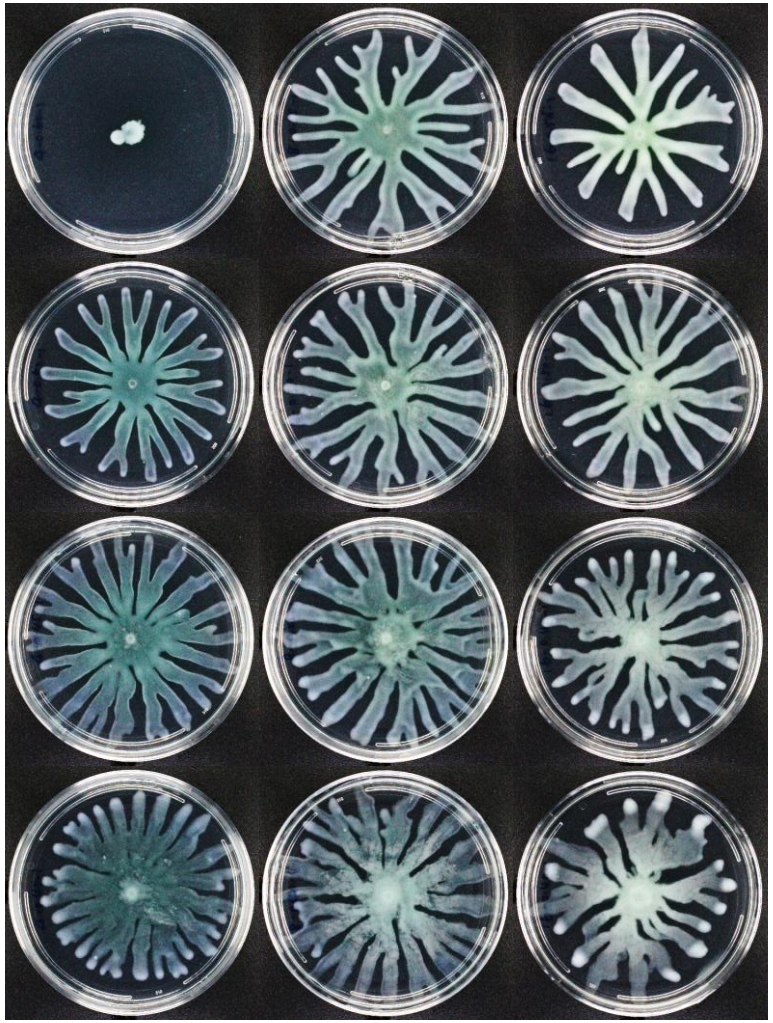

Replicate 4

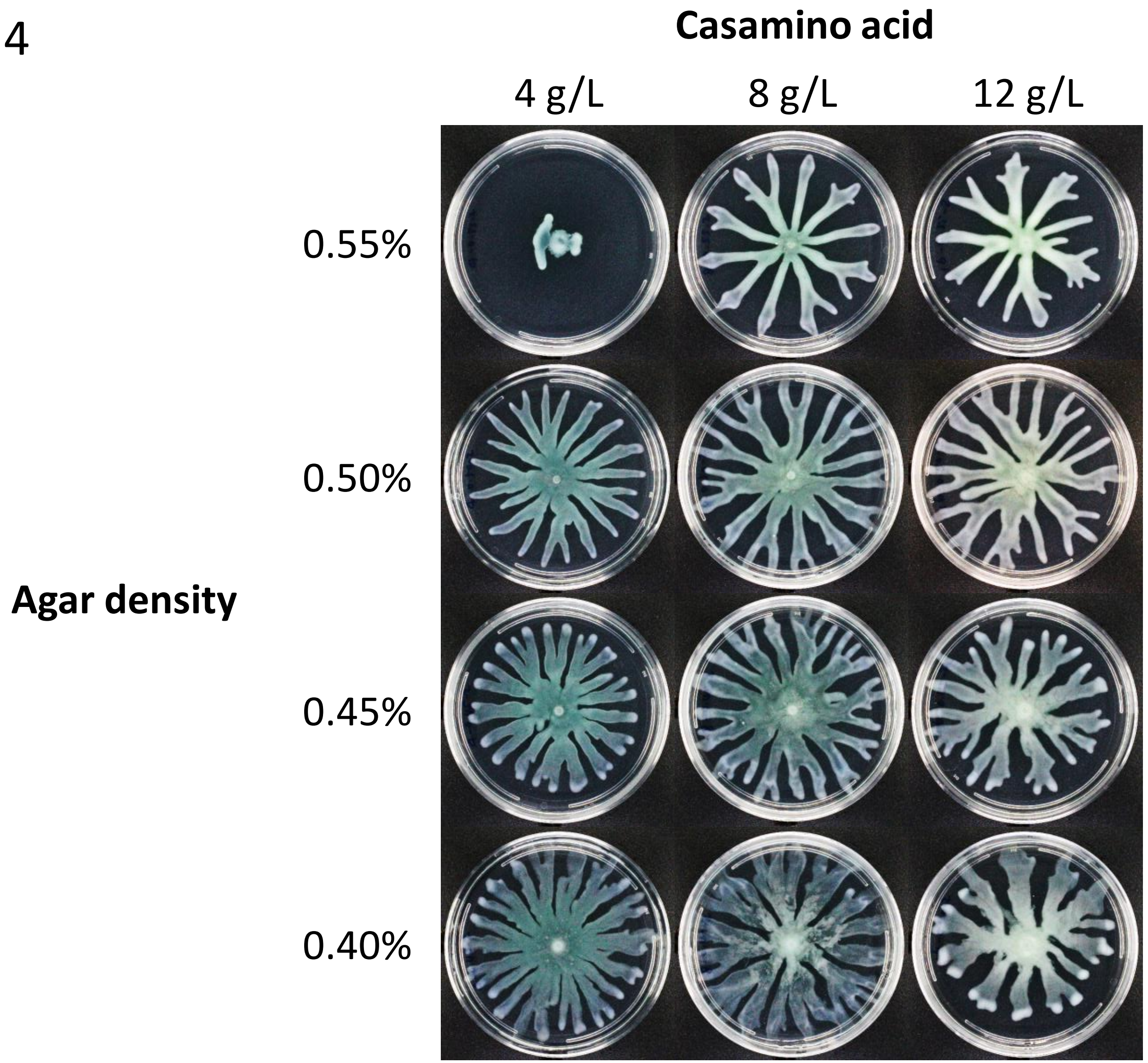

Replicate 5

Casamino acid

5 g/L | 10 g/L

Agar density

0.5%

0.4%

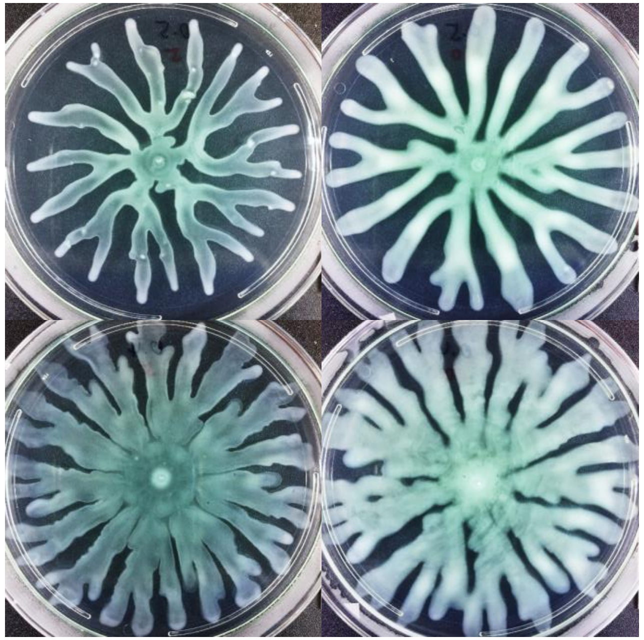

Replicate 6

Casamino acid

5 g/L | 10 g/L | 15 g/L

Agar density

0.55%

0.50%

0.45%

0.40%

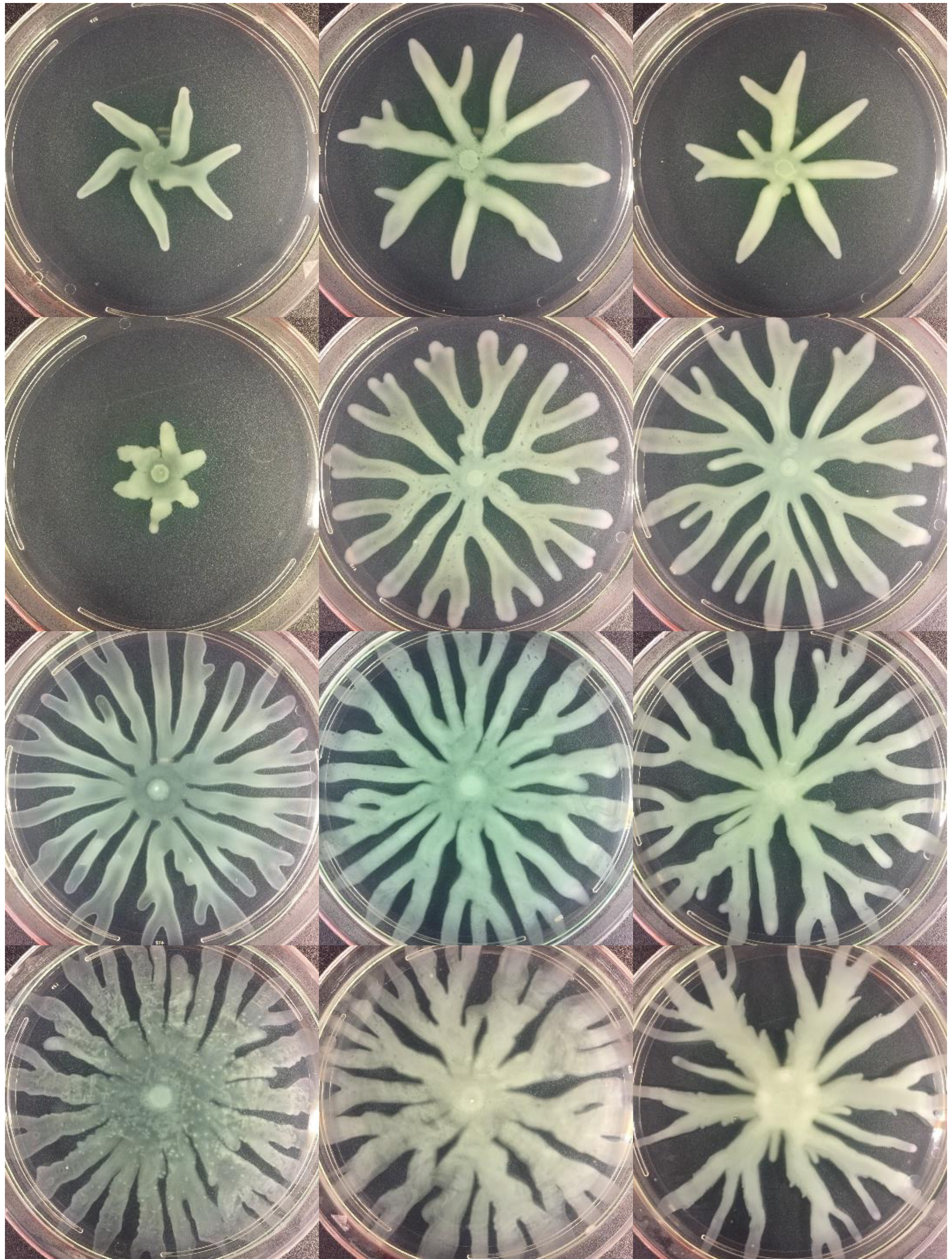

Replicate 7

Casamino acid

5 g/L | 10 g/L | 15 g/L

Agar density

0.55%

0.50%

0.45%

0.40%

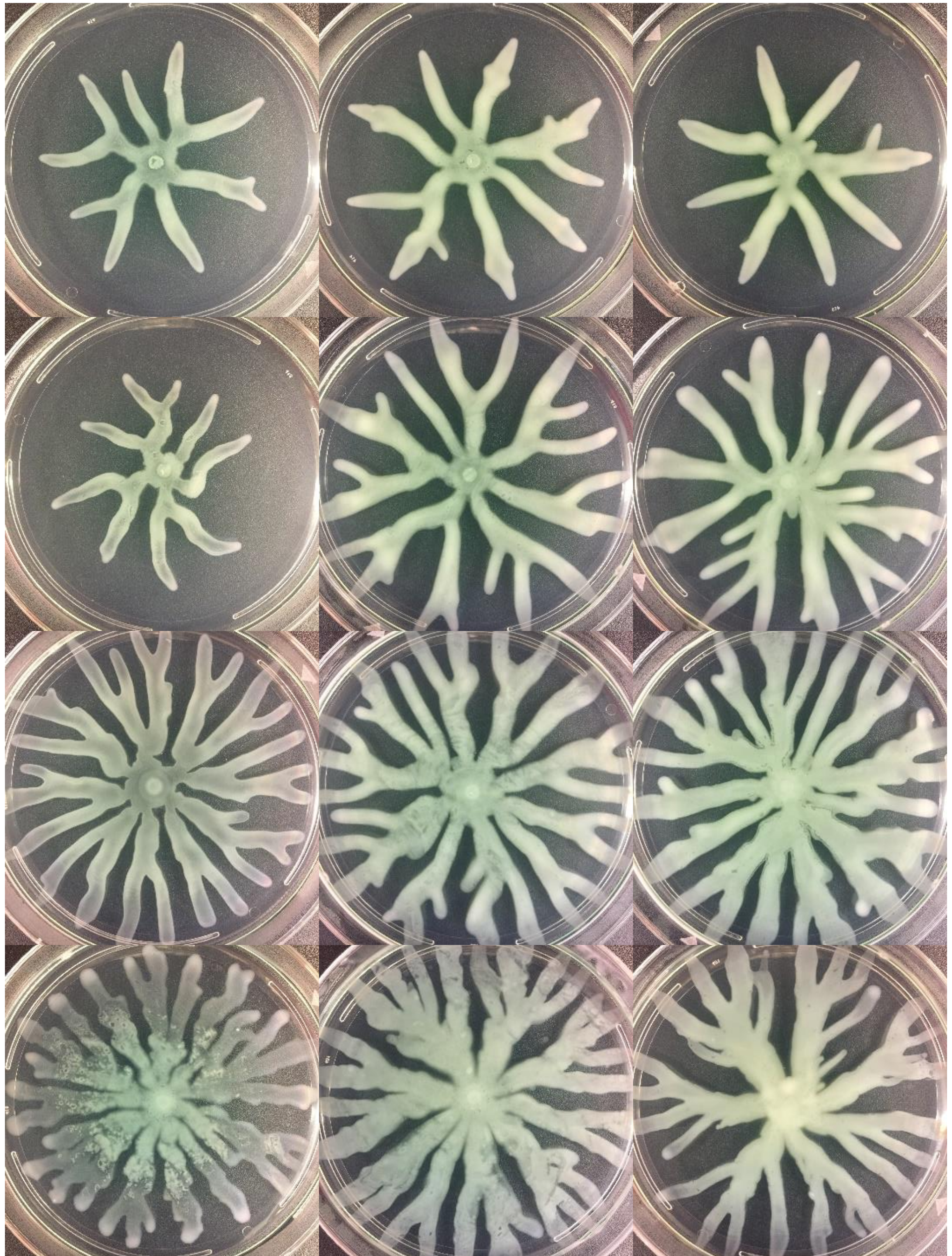

Supplement: Supplementary file 7 — Source Data for Figure 3 [file MSB-17-e10089-s008.zip › Source data for Figure 3/Figure 3 replicates.pdf]
